# Calcineurin Interacts with PERK and Dephosphorylates Calnexin to Relieve ER Stress in Mammals and Frogs

**DOI:** 10.1371/journal.pone.0011925

**Published:** 2010-08-05

**Authors:** Mariana Bollo, R. Madelaine Paredes, Deborah Holstein, Nadezhda Zheleznova, Patricia Camacho, James D. Lechleiter

**Affiliations:** 1 Instituto de Investigación Médica Mercedes y Martín Ferreyra (INIMEC CONICET), Córdoba, Argentina; 2 Department of Cellular and Structural Biology, University of Texas Health Science Center at San Antonio, San Antonio, Texas, United States of America; 3 Department of Physiology, University of Texas Health Science Center at San Antonio, San Antonio, Texas, United States of America; Texas A&M University, United States of America

## Abstract

**Background:**

The accumulation of misfolded proteins within the endoplasmic reticulum (ER) triggers a cellular process known as the Unfolded Protein Response (UPR). One of the earliest responses is the attenuation of protein translation. Little is known about the role that Ca^2+^ mobilization plays in the early UPR. Work from our group has shown that cytosolic phosphorylation of calnexin (CLNX) controls Ca^2+^ uptake into the ER via the sarco-endoplasmic reticulum Ca^2+^-ATPase (SERCA) 2b.

**Methodology/Principal Findings:**

Here, we demonstrate that calcineurin (CN), a Ca^2+^ dependent phosphatase, associates with the (PKR)-like ER kinase (PERK), and promotes PERK auto-phosphorylation. This association, in turn, increases the phosphorylation level of eukaryotic initiation factor-2 α (eIF2-α) and attenuates protein translation. Data supporting these conclusions were obtained from co-immunoprecipitations, pull-down assays, *in-vitro* kinase assays, siRNA treatments and [^35^S]-methionine incorporation measurements. The interaction of CN with PERK was facilitated at elevated cytosolic Ca^2+^ concentrations and involved the cytosolic domain of PERK. CN levels were rapidly increased by ER stressors, which could be blocked by siRNA treatments for CN-Aα in cultured astrocytes. Downregulation of CN blocked subsequent ER-stress-induced increases in phosphorylated elF2-α. CN knockdown in *Xenopus* oocytes predisposed them to induction of apoptosis.

We also found that CLNX was dephosphorylated by CN when Ca^2+^ increased. These data were obtained from [γ^32^P]-CLNX immunoprecipitations and Ca^2+^ imaging measurements. CLNX was dephosphorylated when *Xenopus* oocytes were treated with ER stressors. Dephosphorylation was pharmacologically blocked by treatment with CN inhibitors.

Finally, evidence is presented that PERK phosphorylates CN-A at low resting levels of Ca^2+^. We further show that phosphorylated CN-A exhibits decreased phosphatase activity, consistent with this regulatory mechanism being shut down as ER homeostasis is re-established.

**Conclusions/Significance:**

Our data suggest two new complementary roles for CN in the regulation of the early UPR. First, CN binding to PERK enhances inhibition of protein translation to allow the cell time to recover. The induction of the early UPR, as indicated by increased P-elF2α, is critically dependent on a translational increase in CN-Aα. Second, CN dephosphorylates CLNX and likely removes inhibition of SERCA2b activity, which would aid the rapid restoration of ER Ca^2+^ homeostasis.

## Introduction

The ER is a dynamic organelle that plays a critical role in a variety of processes, including Ca^2+^ storage and release, synthesis and folding of proteins, as well as post-translational protein modification. These processes of signaling and biosynthesis are deeply inter-connected [1], [2], [3], [4], [5].

When the load of newly synthesized proteins exceeds the folding and/or processing capacity of the organelle, the ER enters into a stress condition. This activates a signal transduction pathway called the Unfolded Protein Response (UPR) that attempts to restore homeostasis in the ER [6]. An immediate response is the attenuation of protein translation via PERK, which phosphorylates the α subunit of eukaryotic translation initiation factor 2 (eIF2α) [7], [8]. PERK is a type I ER membrane protein with a stress-sensing luminal domain connected by a transmembrane segment to a cytoplasmic-kinase domain. PERK is normally inactive due to the association of its luminal domain with the ER chaperone BiP. During ER stress, BiP is competitively titrated from the luminal domain of PERK by the excess of unfolded proteins [9]. This dissociation causes PERK to undergo homo-oligomerization and trans-autophosphorylation within its cytosolic kinase domain, thereby increasing its activity. Additional changes that promote long-term adaptation are transcriptional up-regulation of ER chaperones and molecules involved in the ER-associated degradation (ERAD). If ER damage is persistent or excessive, an apoptotic response is initiated by either ER specific caspases [10], [11] or by mechanisms related with the mitogen-activated protein kinase JNK or transcriptional activation of C/EBP homologous protein (CHOP) [12], [13].

Maintenance of Ca^2+^ levels in the ER is primarily attained by the activity of SERCAs [14], [15], [16], which pump Ca^2+^ into the ER. These Ca^2+^-ATPases counteract the loss of Ca^2+^ via leaks and the opening of Ca^2+^ release channels [17], [18], [19]. The free Ca^2+^ in the ER is a balance between Ca^2+^ release, uptake and buffering by Ca^2+^-binding proteins in the lumen. Calreticulin (CRT) and CLNX are Ca^2+^ -binding chaperones that reside in the ER [20], [21] and play key roles in modulating SERCA 2b activity [3], [4], [22]. CRT is entirely luminal and CLNX is a type I trans-membrane protein. The carboxy-terminus of each protein is luminal and is responsible for interaction of the lectins with the monoglucosylated form of N-linked glycoprotein during protein folding [20], [23]. In the cytosolic domain of CLNX, three phosphorylated residues have been identified [24] that are implicated in the modulation of the interaction of CLNX with the ribosome [25]. Dephosphorylation of CLNX causes dissociation of the chaperone from the ribosome [25]. Our group identified the carboxy-terminal serine residue 562 in the rat isoform of CLNX as a phosphorylation site capable of controlling SERCA 2b activity. Further, we demonstrated that CLNX phosphorylation acted as a cytosolic switch that regulated Ca^2+^ store refilling [4].

Calcineurin is a Ca^2+^ and calmodulin dependent serine/threonine phosphatase. This heterodimer phosphatase is composed of a catalytic subunit, calcineurin A (CN-A) and a regulatory subunit, calcineurin B (CN-B) [26]. CN-A contains specific domains with regulatory functions, including an amino-terminus domain with catalytic properties, a CN-B binding domain, a calmodulin (CaM) binding domain and finally, an autoinhibitory domain (AI) at the carboxy-terminus [27]. At resting Ca^2+^ levels, the phosphatase is relatively inactive. An increase in intracellular Ca^2+^ activates CN-A through Ca^2+^/CaM binding, which dissociates AI from the catalytic domain [28]. To date, the involvement of Ca^2+^ signaling in a multitude of cellular pathways has been well documented [17]. However, little is known about the role of Ca^2+^ signaling in restoring ER homeostasis, once ER stress has been triggered. Here we reveal that CN plays key roles in restoring ER homeostasis during stress. CN activity boosts the refilling of Ca^2+^ stores so that optimal conditions for protein processing/folding are rapidly reached. CN also directly interacts with PERK to increase its auto-phosphorylation, which helps to attenuate protein translation while homeostasis is being restored. Finally, we show that a knockdown of CN levels in *Xenopus* oocytes results in a decrease of protein synthesis inhibition and a rapid acceleration of apoptosis. Taken together, these data underscore the importance of CN activity in the rescue of cells from ER stress.

## Results

### CLNX is Dephosphorylated during ER Stress by CN

**Figure 1:**
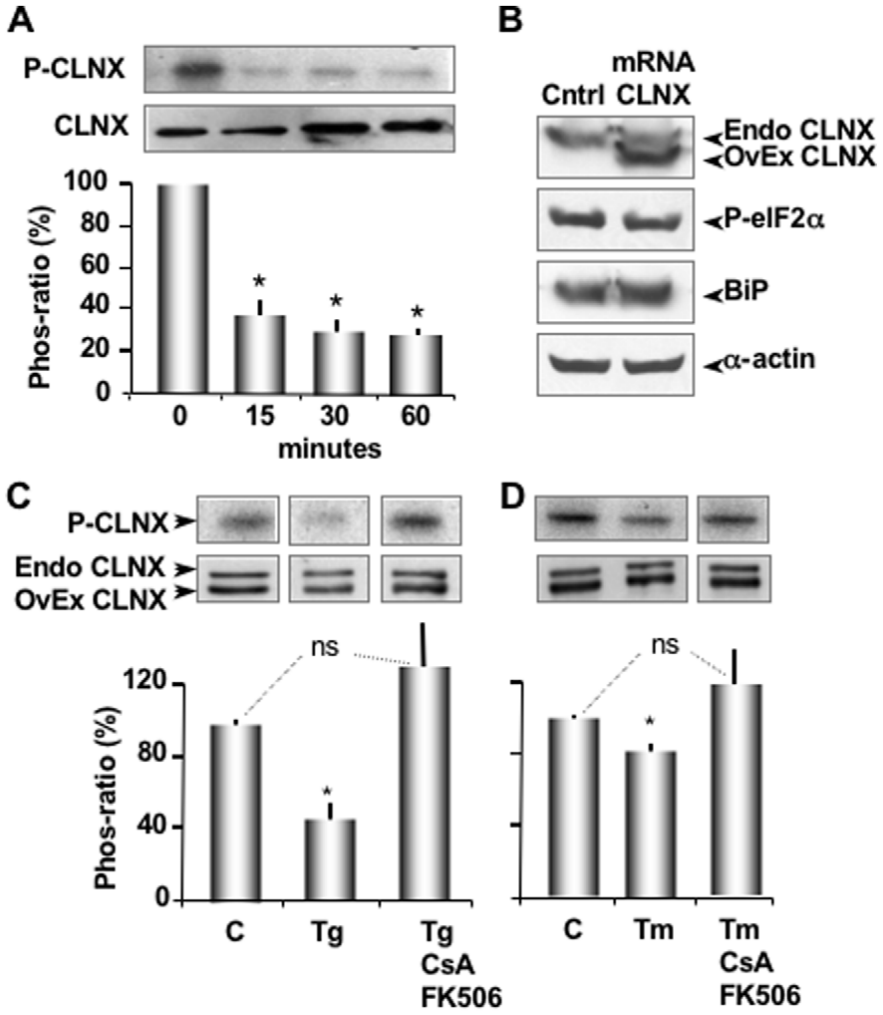
CLNX is dephosphorylated during ER stress by CN. (**A**) IPs of [γ^32^P]ATP-labeled CLNX from oocytes in the absence (0 minutes) or presence (15, 30 and 60 minutes) of Tg (1 µM) were performed. The samples were resolved through 12% SDS-PAGE, transferred to nitrocellulose and P-CLNX visualized by autoradiography (top panel). For loading control, a Western blot of CLNX was performed in oocyte microsomal extracts before the IPs (bottom panel). Histogram depicts the relative intensity of each band relative to the corresponding density of the CLNX Western blot. Notice that exogenous CLNX is expressed at higher levels than endogenous CLNX and that its autoradiographic signal is significantly higher than the signal from endogenous levels of phosphorylatioed CLNX (Figure S1). (**B**) Immunodetection by Western blotting of control oocytes and CLNX overexpressing oocytes. Top panel shows endogenous and exogenous CLNX. Middle panels show phosphorylated eIF2α (P-elF2α) and BiP (Assay Designs cat# SPA-826) in each corresponding cytosolic fraction. Lower panel shows α-actin loading controls. (**C**) Samples from Tg-treated oocytes that were pre-incubated CsA (200 nM) and FK506 (20 nM) for 16 hours are presented in lane 3. Immunodetection of CLNX by Western blotting was used as a loading control (lower panels). Histogram depicts the mean intensity of each band relative to the corresponding density in the Western blots of overexpressed CLNX. DMSO (0.05% v/v) is used as the vehicle control. Notice that control oocytes injected only with CsA/FK506 do not exhibit increased stress as indicated by Western blot analysis of eIF2α -P or BiP (Figure S2). (**D**) Samples from Tm-treated oocytes (lanes 2 and 4) that were pre-incubated or not with inhibitors CsA and FK506 as indicated above are shown in lanes 3 and 4. The middle panels show Western blots of CLNX of the oocyte microsomal extracts before IPs. Histogram shows the relative intensities of P-CLNX compared to overexpressed CLNX. Methanol (0.05%v/v) is used as the vehicle control. Data represents 3 independent experiments with 10 oocytes per group.

Thapsigargin (Tg) is an irreversible inhibitor of the ER Ca^2+^-ATPases [29]. It induces ER stress by depleting Ca^2+^ stores with a concomitant increase in cytosolic Ca^2+^, causing accumulation of malfolded proteins within the ER [30]. By site directed mutagenesis, we previously demonstrated that phosphorylation of serine residue 562 in CLNX controlled an interaction with SERCA 2b. Phosphorylation of S562 inhibited Ca^2+^ store refilling while dephosphorylation increased SERCA 2b activity [4]. Given its ability to regulate SERCA 2b activity, we asked if ER stress altered the phosphorylation state of CLNX. To this end, CLNX mRNA (0.7 µg/µl) was overexpressed in *Xenopus* oocytes as previously described (Roderick et al. 2000). After 3 days of protein expression, oocytes were labelled with [γ^32^P]ATP for 20 minutes, the microsomal fraction was extracted and an anti-CLNX antibody was used to immunoprecipitate the protein. We observed a significant level of phosphorylation of CLNX under normal resting conditions (Figure 1A, 0 minutes). In a subpopulation, we treated CLNX overexpressing oocytes with Tg (1 µM) for 15, 30 and 60 minutes. When the precipitates were examined with autoradiography, we observed significant (p<0.05) dephosphorylation at all time points tested (Figure 1A). To determine if overexpression of CLNX itself caused ER stress, we measured the levels of ER stress in native and overexpressing CLNX oocytes. This was accomplished by a Western blot probed with an antibody that recognizes the phosphorylated form of eIF2α (anti-phospho eIF2α) and an antibody against BiP, both are widely considered strong indicators of ER stress [8], [30]. Overexpression of CLNX did not affect the level of phosphorylated eIF2α or BiP and hence, did not induce ER stress (Figure 1B). These results show that ER Ca^2+^ depletion and/or increased cytosolic Ca^2+^ decreases CLNX phosphorylation. To test whether CN may be mediating the Ca^2+^ sensitive dephosphorylation of CLNX, we repeated the above series of experiments using the CN inhibitors cyclosporin A (CsA) and FK506. Preincubation of oocytes with these inhibitors completely reversed the dephosphorylation of CLNX in response to Tg (Figure 1C) and treatment consistent with a primary role of CN in this ER stress response.

### ER Ca^2+^ Release is Implicated In CLNX Dephosphorylation by CN after Tunicamicyn Treatment

Tunicamycin (Tm) has a different mechanism of action than Tg to induce ER stress. It inhibits glycosylation of nascent proteins thereby causing accumulation of malfolded proteins in this organelle [31]. To determine whether this ER stressor also leads to dephosphorylation of CLNX, *Xenopus* oocytes overexpressing CLNXs and labeled with [γ^32^P]ATP as described above were used. As with Tg, Tm treatment of overexpressing oocytes significantly (p<0.05) induced dephosphorylation of CLNX. Similarly, CN inhibitors cyclosporin A and FK506 completely reversed this stress induced dephosphorylation (Figure 1D).

### Tm Treatment Increases Cytosolic Ca^2+^

**Figure 2:**
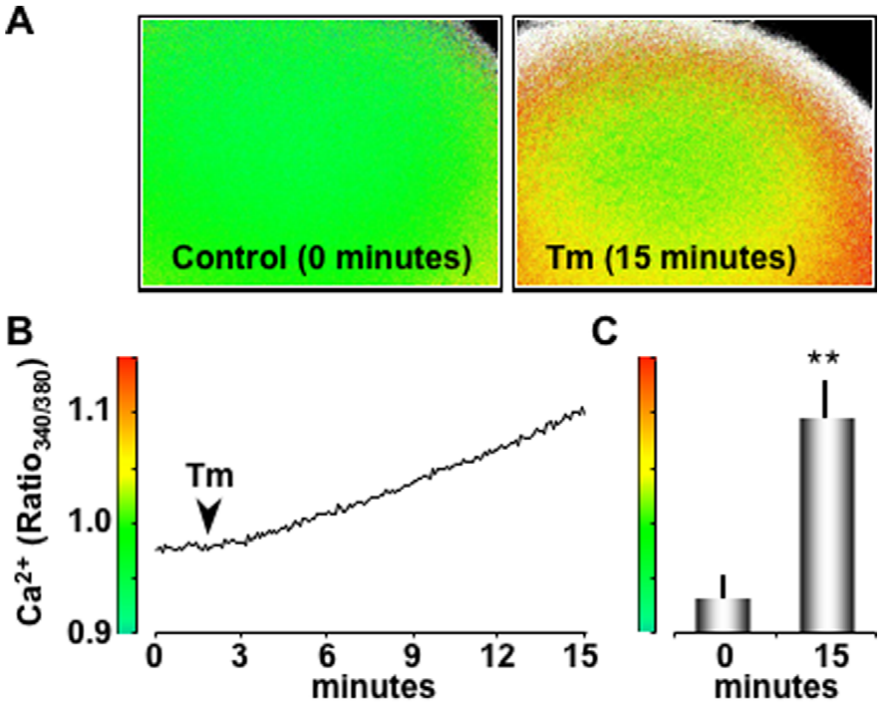
Tm treatment increases cytosolic Ca^2+^. (**A**) Images of Fura-2 loaded oocytes before (0 minutes) and after (15 minutes) Tm treatment. Ca^2+^ levels are presented as fura-2 fluorescence ratios of 340 to 380 nm excitation. The intensity scale bar for these images is presented in B and C. (**B**) Time course of Fura-2 ratio (Ratio_340/380_) changes in response to Tm treatment (2.5 µg/ml, added at arrow). (**C**) Histogram of the average Ratio_340/380_ (n = 9 oocytes, pooled from 3 independent experiments) at rest (0 minutes) and after Tm treatment (15 minutes).

The dependence of CLNX phosphorylation on CN activity suggested that Ca^2+^ was being released into the cytosol during Tm-induced ER stress. To test this hypothesis, we measured cytosolic Ca^2+^ in single oocytes using fluorescence microscopy. *Xenopus* oocytes were injected with the ratiometric Ca^2+^ indicator dye Fura 2 (50 µM final concentration, Invitrogen-Molecular Probes, Eugene, OR). After a 20–30 minutes, oocytes were imaged. Ca^2+^ levels were expressed as the ratio of fluorescence for 340 and 380 excitation (R_340/380_). When Fura-2 loaded oocytes were exposed to Tm (2.5 µg/ml), we observed a slow rise in cytosolic Ca^2+^ (Figure 2). The average resting Fura-2 ratio was 0.93±0.02 (n = 9 oocytes), which corresponded to 129±6 nM with *in vitro* calibration. After 15 minutes of Tm bath incubation, the Fura-2 ratio was significantly (p<0.04) increased to 1.09±0.03 corresponding to 174±9 nM Ca^2+^. In parallel experiments, we also found that treatment with Tg (1 µM) for 15 minutes increased the Fura-2 ratio to 1.01±0.04 (n = 11 oocytes), corresponding to 150±10 nM Ca^2+^. These data suggest that Tm treatment releases Ca^2+^ from intracellular stores.

### CN Interacts with PERK in a Ca^2+^ Dependent Manner

**Figure 3:**
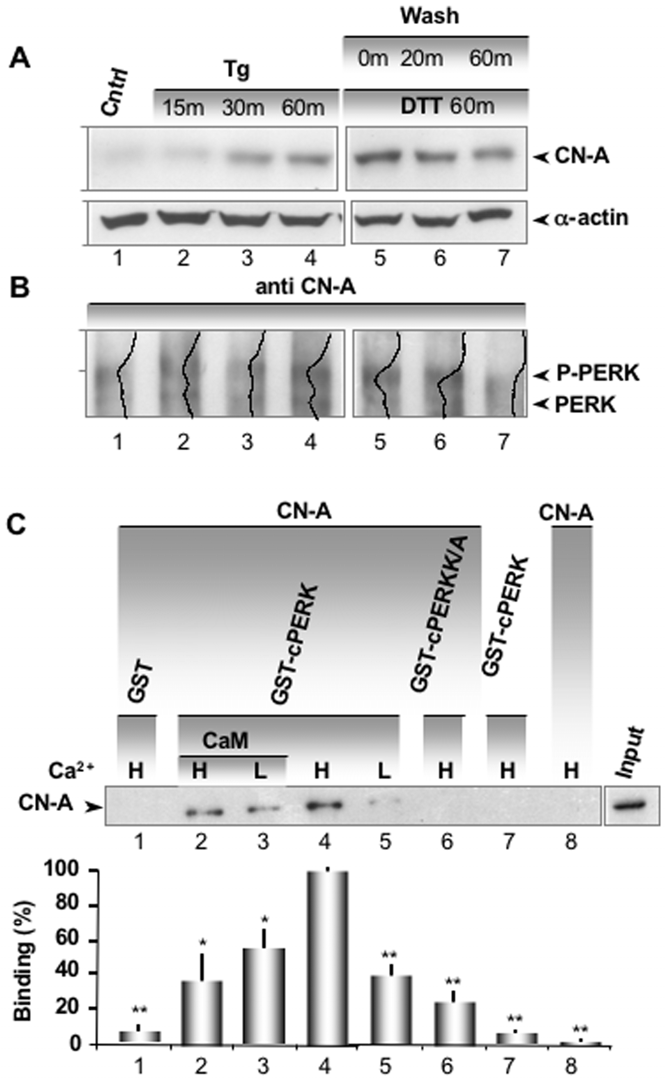
CN interacts with PERK and is Ca^2+^ dependent. (**A**) Immunodetection (Western blot) of CN-A in cytosolic extracts obtained from control oocytes (lane 1), oocytes treated with Tg for 15, 30 or 60 minutes (lanes 2, 3 and 4, respectively), or oocytes treated with DTT for 60 minutes and washed for 0, 20 or 60 minutes (lanes 5, 6 and 7, respectively). The samples were resolved through 12% SDS-PAGE and transferred to nitrocellulose (loading 0.2 oocyte equivalents per lane). α-actin loading controls are presented for the corresponding extract in the bottom panel. The samples correspond to the same experiment but were run on two separate gels with equal exposure times. Note also that lane 1 is the untreated control for both Tg and DTT treated oocytes. (**B**) Co-IP between CN-A and PERK corresponding to the same treatments (lanes) presented in **A**. The samples were resolved through 7% SDS-PAGE, loading the immunoprecipitate from an input of 20 oocytes per lane and transferred to nitrocellulose. The IP was performed first with anti CN-A antibody and was followed by immunodetection by Western blot with anti-PERK antibody. A line profile (Image J, NIH) of each lane is overlayed to highlight the distribution of the main peaks corresponding to the two variants of PERK (P-PERK is retarded with respect to PERK). Note the increased level of P-PERK in oocytes stressed for 60 minutes with Tg (lane 4) and DTT (lane 5). The level of P-PERK returned to normal levels after 60 minutes of wash (lane 7). The experiment was repeated 4 times. Changes in CN expression and PERK phosphorylation were only observed in response to ER stress when the resting level of CN was low (lane 1), indicative of initially unstressed oocytes. CN-A levels of the IP presented in Figure 3B are showed in Figure S3. We demonstrate the IP efficiency and specificity for PERK, and ruled out non-specific binding of PERK to beads (Figure S4 A, B). Moreover, we generated a new antibody for PERK and we present a characterization of its specificity in Figure S4 C–E. We show that the new antibody, labeled anti-PERK^UT^, recognizes a protein band around the expected molecular weight of PERK (150 kD) and that the antibody is competed off by incubation with the antigen peptide that was used to generate the antibody. (**C**) GST pull-down assay between CN-A α/B and GST-cPERK, at low Ca^2+^ (L = 46 nM) and high Ca^2+^ (H = 3.2 µM). CN-A pull-down levels are shown for GST alone (lane 1), GST-cPERK in the presence (lanes 2 and 3) and absence (lanes 4 and 5) of CaM for high and low Ca^2+^, GST-cPERK K/A in high Ca^2+^ (lane 6), GST-cPERK without CN-A (lane 7) and CN-A without GST-cPERK (lane 8). The proteins were incubated with glutathione sepharose 4B for 1 hour followed by boiling in Laemmli reducing Buffer, resolved through 12% SDS-PAGE followed by Western blotting using a monoclonal mouse anti CN-A. The Western blot on the panel right of lane 8 indicates the CN-A input. We calibrated the loading of GST-cPERK and GST-cPERK K/A using an albumin standard curve (Figure S5). This insured that equal molar amounts of protein were loaded in each lane. Histogram corresponds to densitometric analysis from the average of these experiments. One asterisk corresponds to a statistical significant difference (p<0.05, ANOVA test, n = 4 independent experiments) and two asterisks denote a statistical significant difference of (p<0.001; ANOVA test, n = 11 independent experiments) using Lane 4 as 100% control value.

Our data revealed that CN dephosphorylation of CLNX occurred rapidly in response to ER stress. Since PERK is presently considered the most proximal luminal sensor of the UPR [8], we wondered if there was a functional relationship between CN and PERK. To initially address this question, *Xenopus* oocytes were treated with Tg (1 µM) for either 15, 30 or 60 minutes. A second group of oocytes were initially treated with DTT (1 mM) for 60 minutes and then washed for either 0, 20 or 60 minutes. Like Tg and Tm, DTT is an ER stress inducer, but its effects are reported to be reversible [9], [32]. Protein extracts were prepared from each of the six groups of oocytes along with an untreated, control group. The cytosolic fractions were run on SDS-PAGE and analyzed by Western blot with anti-CN-A antibody (Figure 3A). We observed that the expression level of CN-A increased significantly after 30 and 60 minutes following Tg treatment and also after 1 hour of treatment with DTT (Figure 3A). Partial reversal of the 1 hour exposure of oocytes to DTT was obtained by washing the treated oocytes for another hour before preparing the protein extract. Furthermore, we examined whether endogenous CN could associate with the endogenous PERK by co-immunoprecipitations (Co-IPs) of CN-A with PERK in the same oocytes stressed with either Tg or DTT as presented above (Figure 3B). The respective microsomal fractions were immunoprecipitated with anti-CN-A antibody, run on SDS-PAGE and analyzed by Western blot with anti-PERK antibody, which labeled both phosphorylated and the higher mobile unphosphorylated PERK. First, we found that the largest amount of CN-A that co-immunopurified with P-PERK/PERK occurred at the highest level of ER stress (60 minutes) for both Tg or DTT treatment (Figure 3A and 3B, lanes 4 and 5). Second, the presence of CN-A appeared to increase PERK phosphorylation levels. We note that PERK runs as heterogenous population depending on its level of phosphorylation, since it has been shown to have at least 10 phosphorylation sites [33]. The CN-A/P-PERK/PERK interaction returned to the control levels of unstressed oocytes (Figure 3B, lane 1) after a 60-minutes washout of DTT (Figure 3B, lane 7). We conclude from these data that under ER stress CN-A interacts with PERK and this association appears to increase phosphorylation of PERK.

Given evidence for a functional interaction between CN-A and PERK, we asked if there was a physical interaction between these proteins. We also tested whether Ca^2+^ and calmodulin (CaM) affected this interaction given the known dependence of this phosphatase on Ca^2+^ and CaM. To this end, *in vitro* GST pull-down experiments were performed between PERK and CN-A, using two Ca^2+^ concentrations that were chosen to mimic high (H, 3.2 µM) and low (L, 30 nM) cytosol levels. A GST fusion protein was created with only the cytosolic domain of PERK (GST-cPERK), which was then used to pull down recombinant human CN-A α and CN-B. We found that the interaction of CN-A with GST-cPERK was significantly (p<0.01) stronger in high Ca^2+^ concentration (Figure 3C, lane 4 vs lane 5) and that this interaction was decreased by calmodulin (CaM), irrespective of the Ca^2+^ concentration (Figure 3C, lanes 2 and 3) (p<0.05). CN-A and CaM were used at equimolar concentrations for this experiment and there was no significant binding of CN-A to GST alone or to glutathione-sepharose (Figure 3C, lanes 1 and 8, respectively). We also created a GST fusion construct with an inactive PERK kinase mutant where lysine 618 was mutated to alanine (GST-cPERK K/A) [8]. Interestingly, the inactive PERK mutant lacked significant binding to CN-A (Figure 3C, Lane 6). Together these findings corroborate our previous observation that CN-A specifically binds to purified, fully active, cytosolic PERK. This association does not appear to be mediated by another protein and the interaction is strongest in high Ca^2+^, conditions that would be expected to occur immediately after ER stress is first induced.

### PERK Auto-Phosphorylation Increases with the Interaction of CN-A and PERK

**Figure 4:**
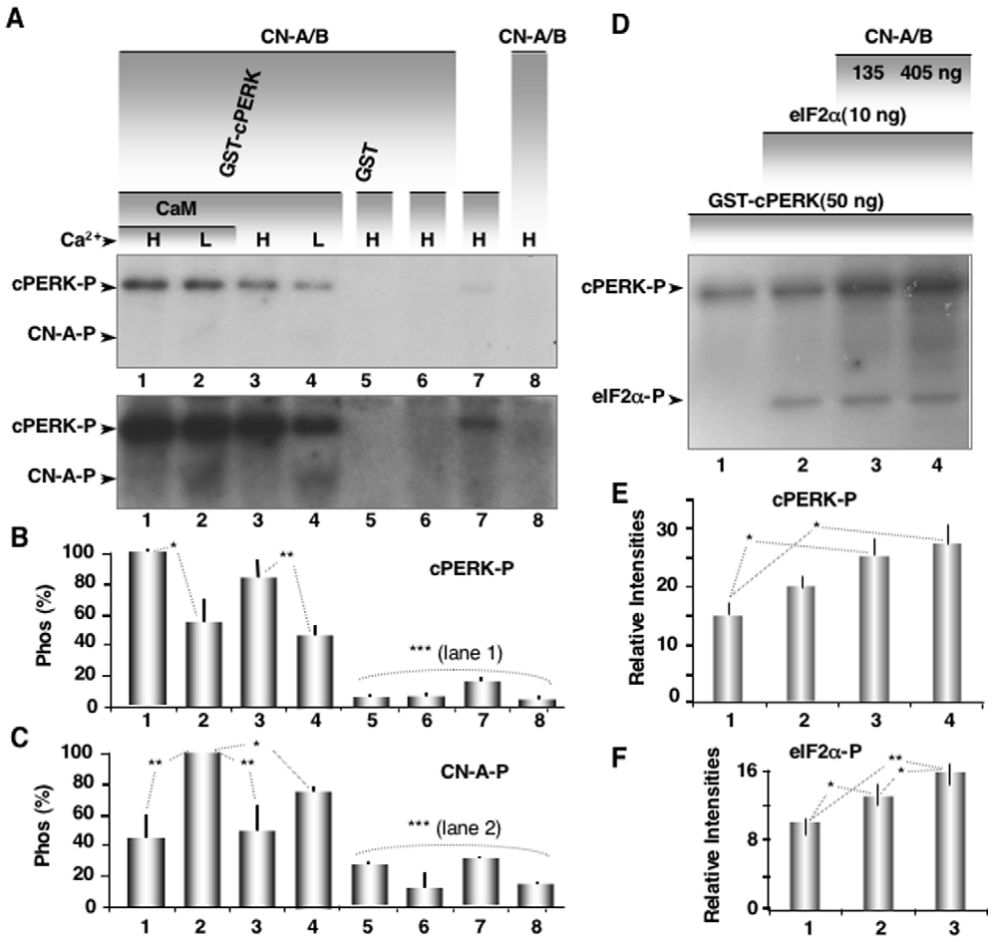
PERK auto-phosphorylation and kinase activity increases with the interaction of CN-A and PERK. (**A**) GST-cPERK and CN-Aα/B were incubated with [γ^32^P]ATP, resolved through 12% SDS-PAGE and visualized by autoradiography as described in Materials and Methods. Phosphorylation levels are shown for GST-cPERK in the presence (lanes 1 and 2) and absence (lanes 3 and 4) of CaM for high (H, 1.2 µM) and low (L, 30 nM) Ca^2+^, for GST-alone (lane 5), for GST-cPERK K/A in high Ca^2+^ (lane 6), for GST-cPERK without CN-A (lane 7) and for CN-A α/B without GST-cPERK (lane 8). Histogram corresponding to densitometric analysis of cPERK auto-phosphorylation (**B**) or CN-A phosphorylation (**C**) from the average of three independent experiments (n = 3), using as 100% control value lane 1 in **B** and lane 2 in **C,** respectively. See the Commassie blue gel for the loading control of the autoradiogram (Figure S6). (**D**) Kinase assay was performed as described above in the presence of 2 µM Ca^2+^, but adding increasing amounts of CN-Aα/B and in the presence of eIF2α (50 nM). Histogram corresponding to densitometric analysis of cPERK auto-phosphorylation (**E**) or CN-A phosphorylation (**F**) from three independent experiments (n = 3). One asterisk corresponds to a statistical significant difference (p<0.05, ANOVA test) and two asterisks denote a statistical significant difference of (p<0.001; ANOVA test).

To further investigate the CN-A/PERK interaction, we performed *in vitro* kinase assays. GST-purified proteins were incubated with a phosphorylation reaction mixture containing [γ^32^P]ATP. From this assay, we uncovered three important findings. First, autophosphorylation of PERK was significantly increased in the presence of CN-A (Figure 4A and B lanes 1–4 vs lane 7). Second, CN-A itself was phosphorylated at low Ca^2+^ concentrations (Figure 4A and C, lanes 2 and 4 vs lanes 1 and 3). And third, PERK phosphorylation was significantly less in low Ca^2+^ concentrations, when CN-A was phosphorylated. CN-A was not phosphorylated in the absence of GST-cPERK (Figure 4A, lane 8) or with the kinase mutant GST-cPERK K/A (Figure 4A, lane 6) or with GST alone (Figure 4A, lane 5). In addition, there was no phosphorylation of the 19 kD regulatory subunit of CN-B, which was included in the reaction mixture. These data confirm the results suggested in Figure 3B, that the interaction of CN-A with cPERK increases autophosphorylation of the kinase. They also demonstrate for the first time, to our knowledge, that *in vitro* CN-A is a PERK substrate at low Ca^2+^ concentrations and that PERK autophosphorylation is reduced when CN-A is phosphorylated.

We wanted to examine the functional consequences of the promoting cPERK autophosphorylation (Figure 4D). Adding increasing amounts of CN-A α/B increase cPERK autophoshorylation as well as PERK-mediated eIF2α phosphorylation (Figures 4E and 4F). We also wanted to determine if CN-A phosphorylation affected its phosphatase activity. To accomplish this, we setup a spectrophotomeric assay that measured the enzyme activity of recombinant phosphorylated and nonphosphorylated CN-A/B at high (1.4 µM) and low (40 nM) Ca^2+^ concentrations. Indeed, phosphorylated CN exhibited significantly lower specific activity than unphosphorylated CN (CN-ATP) or CN combined with PERK (CN-PERK) and P-CN enzyme activity was further diminished in low Ca^2+^ (Figure S7). Finally, the V_max_ for phosphorylated and non-phosphorylated CN-A was significantly different at low Ca^2+^ while the Km did not change (Table S1). We concluded from these experiments that the phosphatase activity of CN-A is significantly diminished by phosphorylation. Together, these data suggest a new feedback loop that would further enhance recovery from ER stress. As Ca^2+^ levels decrease, CN-A becomes phosphorylated, which further reduces its activity and helps to shutdown ER stress.

### Knock-Down of CN-A Attenuates Protein Synthesis Inhibition during ER Stress

**Figure 5:**
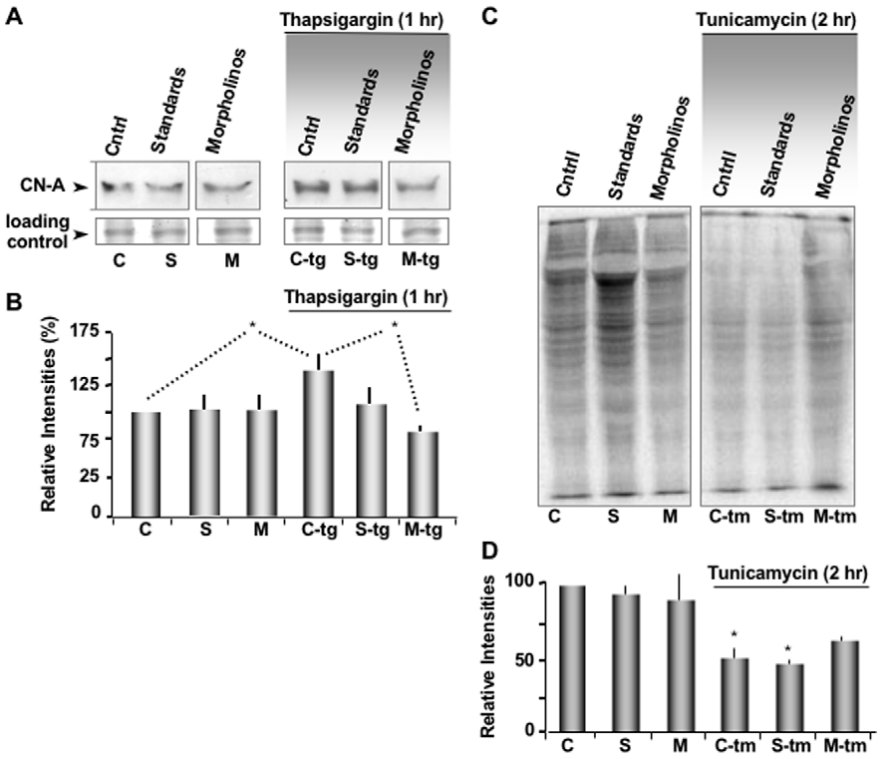
Knockdown of CN-A attenuate the protein synthesis inhibition under ER stress. (**A**) CN-A expression levels are shown for the following conditions: control oocytes (lanes C and C-tg)(Cntrl, lane 1), oocytes injected with standard control morpholinos (lanes S and S-tg) (Std Morpho, lane 2), oocytes injected CN-A morpholinos (lanes M and M-tg) (Morpho CN 1& 2, lane 3). A subgroup of each oocyte pool was also stressed for 30 minutes with Tg (lanes C-tg, S-tg and M-tg 4–6). Note that CN is decreased by the CN-A morpholinos treatment only after ER stress (lane M-tg 6). Expression levels are indicated by Western blot (top panels). Loading controls are presented in the bottom panels. All bands were from the same gel and received the same exposure time. (**B**) Histogram depicts the mean intensity of each band relative to the corresponding density in the Western blots of CN. Data pooled from three independent experiments (n = 3). (**C**) Autoradiography of total protein synthesized in control oocytes or injected with morpholinos as was described above, that has been untreated or exposed to Tm before a 45 minutes pulse label with [^35^S]-Methionine-Cysteine. The two panels are from the same gel and received the same exposure time. (**D**) Histogram corresponding to densitometric analysis of total protein. Data pooled from three independent experiments (n = 3).

To assess the physiological significance of the CN/PERK interaction, we compared protein synthesis rates after knocking down CN and treatment with ER stressors. Test oocytes were injected with two different morpholino antisense oligonucleotides specific for *Xenopus* CN-A mRNA to inhibit the expression of this protein. Morpholino treatments, rather than interference RNA techniques are required to knockdown protein expression in *Xenopus* oocytes [34], [35]. Oocytes were injected with morpholino oligonucleotides (Morpho CN 1&2) and CN-A expression was analyzed by Western blot. We observed no significant effect on resting levels of CN expression within 2 hours of the initial morpholino injection (Figure 5A). However, when oocytes were treated with the ER stressor Tg (1 µM, 30 minutes), the previously observed increase in CN level (Figure 3A) was blocked. Protein synthesis measured by pulsing cells with [^35^S]-Methionine-Cysteine showed no significant changes in oocytes injected with either CN morpholinos or standard control oligos as well as uninjected control oocytes. However, we observed an expected reduction after treatment with Tm (Figure 5C) and Tg (Figure S8). This inhibition of protein translation was significantly attenuated by knocking down CN. This experiment establishes a strong correlation between CN-PERK interaction and protein synthesis inhibition under ER stress.

### CN-A Levels are Rapidly Increased in Astrocytes During ER Stress and are Required for Stress-Induced Increases in Phosphorylated elF2α

**Figure 6:**
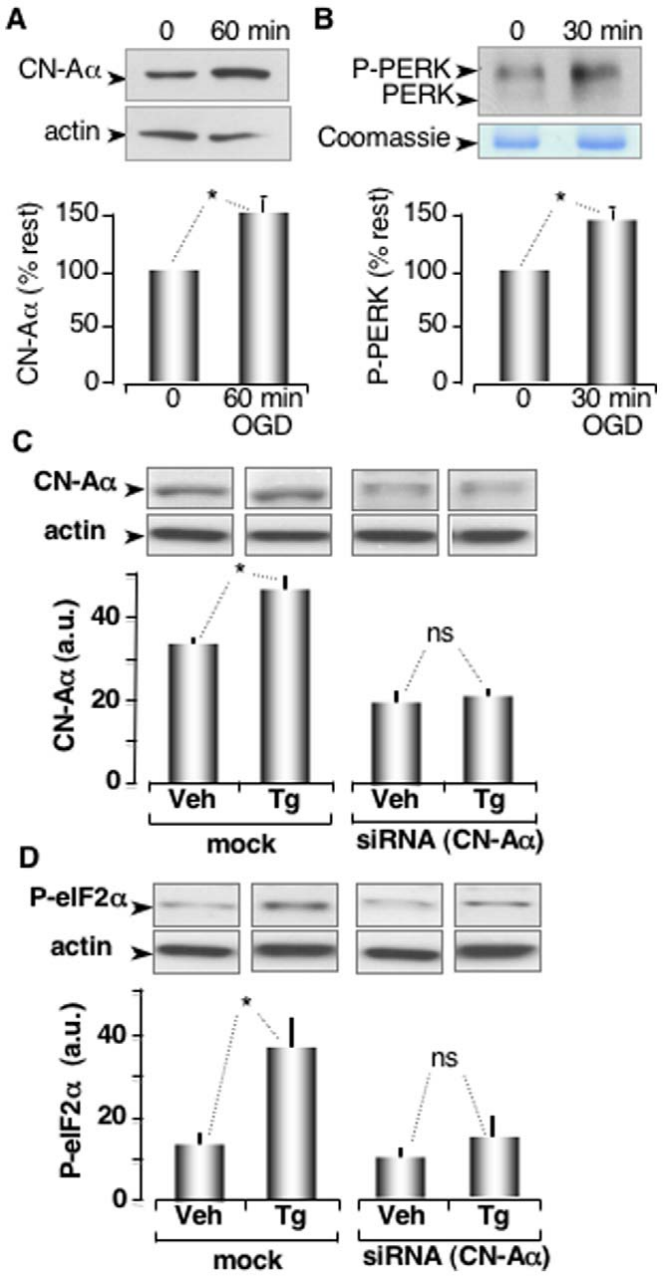
Stress-induced increases in CN-Aα levels enhance phosphorylation of PERK and elF2α. (**A**) Western blot analysis of CN-Aα levels before and after 60 minutes of OGD treatment. Astrocyte cytosolic extracts were resolved on a 12% SDS-PAGE, transferred to nitrocellulose and probed with anti CN-A antibody (Assay Designs cat# SPA-610). A densitometry histogram normalized with actin levels is presented below (n = 5, p<0.05). (**B**) Co-IP between CN-Aα and PERK corresponding to untreated cells (0 minutes) and OGD treated (30 minutes). The samples were resolved on a 7% SDS-PAGE by loading the CN-Aα immunoprecipitate from astrocytes and transferred to nitrocellulose. The IP was performed with the same anti CN-A antibody, followed by a Western blot with anti PERK antibody (ABGENT cat# AP8054b). A sample from the immunoprecipitate was stained with Coomassie as loading control. Densitometry histogram normalized with Commassie (n = 4, p<0.05). (**C**) Western blot analysis of CN-Aα levels in astrocytes transfected with siRNA or reagents only (mock) and subsequently treated with vehicle (Veh) or thapsigarin (Tg) for 1 hour. Densitometry histogram is normalized with actin (n = 4, p<0.01). (**D**) Western blots of astrocyte extracts probed with anti P-eIF2α antibody. Densitometry histogram is normalized with actin (n = 4, p<0.01).

Data obtained in *Xenopus* oocytes indicated that stress-induced increases in CN-A levels enhanced PERK autophosphorylation and the subsequent attenuation of protein synthesis inhibition. To determine if CN-A also regulated PERK activity in another model system, we exposed cultured astrocytes to oxygen glucose deprivation (OGD), an *in vitro* model of ischemia. We observed a significant increase in the levels of CN-A (Figure 6A). Co-immunoprecipitation experiments also revealed that CN-A bound to PERK and that the phosphorylated level of PERK was significantly higher after 30 minutes of OGD (Figure 6B).

To ascertain whether stress-induced increases in CN-Aα were dependent on translation, we treated cultured astrocytes with siRNA specific for CN-Aα for 24 hours. Western blot analysis revealed that CN-Aα levels were significantly reduced by ∼50% (Figure 6C). When these siRNA treated astrocytes were exposed to thapsigargin (Tg) for 1 hour, CN-Aα levels were not significantly affected, whereas control, mock-transfected astrocytes exhibited the normal CN-Aα increase (Figure 6C). We conclude from these data that Tg-induced increases in CN-A levels are likely due to enhanced translation.

We further tested the impact of Tg-induced increases in CN-Aα on the UPR, as indicated by phosphorylation of elF2α (P-elF2α). siRNA (CN-Aα) treated astrocytes showed no significant increase in P-elF2α in response to Tg treatment. Control astrocytes that were mock-transfected exhibited expected increase in P-elF2α (Figure 6D). We conclude from these data that Tg-induced increases in CN-A levels significantly enhance phosphorylation of elF2α. Together, these data suggest that the early UPR induced by ER stress is critically dependent on a rapid increase in CN-Aα.

### Knock-Down of CN-A Enhances Apoptosis in *Xenopus* Oocytes

**Figure 7:**
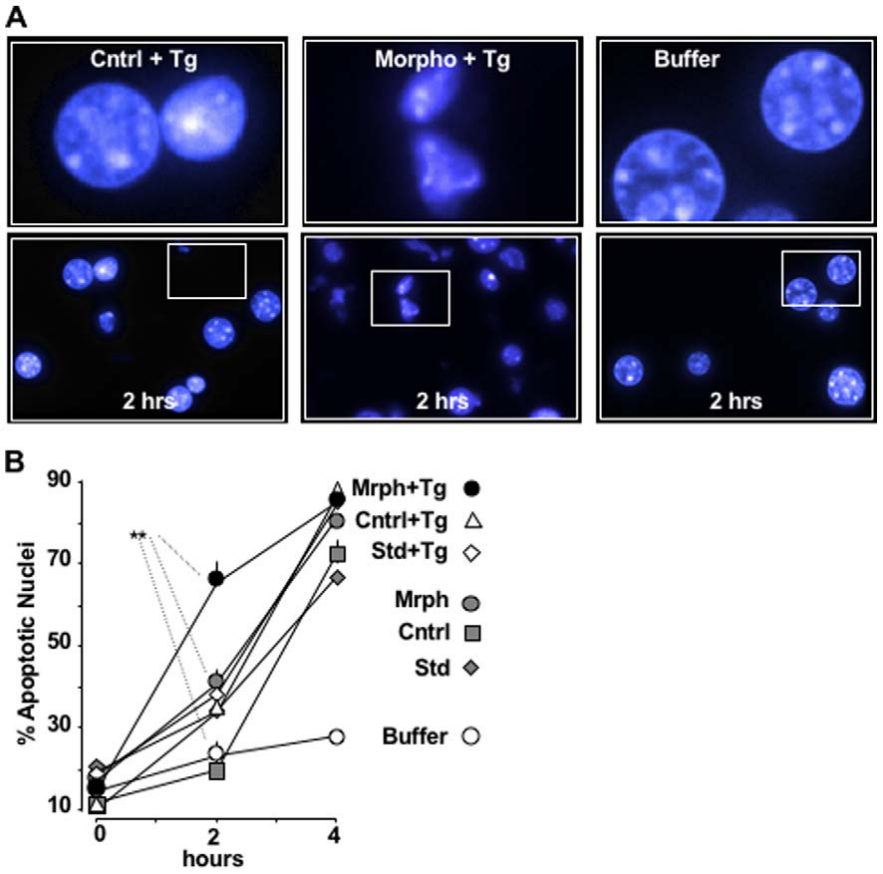
Knockdown of CN-A increases the appearance of apoptotic nuclei in *Xenopus* oocyte extracts. (**A**) Apoptotic potency of cytosolic extracts obtained from control oocytes treated with Tg (Cntrl + Tg) or from oocytes injected with CN-A morpholino 1 & 2 treated with Tg (Morpho CN 1&2 + Tg) compared with buffer alone. Images of liver nuclei were obtained at 2 hours. Note the large number apoptotic-like nuclei at 2 hours for Morpho + Tg oocyte extract. (**B**) Lineplot of the average of the percentage of apoptotic nuclei at 0, 2 and 4 hours for cytosolic extract obtained from control oocytes with and without Tg treatment (Cntrl, Cntrl + Tg), from oocytes injected with standard morpholino with and without Tg treatment (Std Morpho, Std Morpho + Tg) and from oocytes injected with CN-A morpholino oligos 1 & 2 with and without Tg treatment (Morpho CN1&2, Morpho CN1&2+ Tg), compared to nuclei incubated with buffer alone. Data were obtained from 4 independent experiments in which 150 oocytes per group were used for each condition. **p<0.01.

Given the fact that cells commit to cell death if they are unable to reduce or recover from ER stress, we wanted to test the physiological impact of CN activity on apoptosis. To accomplish this, we took advantage of an assay originally pioneered by Newmeyer and co-workers [36] and modified by our laboratory to work with *Xenopus* oocytes [37]. We found that oocyte extracts contained all of the molecular machinery necessary to induce apoptotic-like morphological changes in isolated liver nuclei that were added to the mixture. For this assay, immature oocytes were lysed and centrifuged to remove yolk and lipids. The remaining cytosolic extract was mixed with liver nuclei, which were stained with Hoechst dye at 0, 2 or 4 hours to score for apoptotic morphology. The percentage of nuclei exhibiting apoptosis reached a maximum approximately 4 hours after initial exposure to cytosolic extract, whereas no significant changes were observed in buffer treated nuclei (Figures 7A, S9). Cytosolic extract was then prepared from oocytes injected with either CN morpholinos (oligos 1 and 2) or standard control oligos as well as uninjected control oocytes to determine how CN activity affected apoptosis. A subpopulation of oocytes from each group was also treated with Tg (1 µM, 30 minutes). The apoptotic potency of each extract was assayed at 0, 2 and 4 hours. We found that cytosolic extract prepared from Tg-stressed oocytes previously injected with CN morpholino oligos (Morpho + Tg) exhibited a significantly (p<0.01) rapid increase in apoptosis at 2 hours compared to control Tg-stressed oocytes (Cntrl + Tg) or to buffer alone (Figure 7 A–B). We conclude from these results that the rapid expression of CN-A subsequent to ER stress, delays cells from undergoing apoptosis. This suggests that one of the physiological functions of CN immediately post-ER stress is to protect cells, giving them time to recover and restore ER homeostasis.

## Discussion

In this study, we have shown that CN works to restore ER homeostasis immediately after ER stress has been initiated. CN performs this important function with the aid of two ER transmembrane proteins: CLNX and PERK. Consequently, CN can now be viewed as an active participant in the UPR by virtue of its ability to couple the cytoplasmic side to the ER lumen in a Ca^2+^ dependent manner. We previously established that when the ER is optimally loaded with Ca^2+^, the most favorable condition necessary for protein processing and folding, CLNX is phosphorylated and physically interacts with SERCA 2b to inhibit its activity [4]. We also demonstrated that IP_3_-mediated Ca^2+^ release caused a Ca^2+^ dependent dephosphorylation of serine residue (S562) in the cytosolic domain of CLNX. This removed the functional interaction of CLNX with the pump, removing inhibition and maximizing SERCA 2b-mediated Ca^2+^ store refilling. CLNX phosphorylation had already been shown to regulate its association with the ribosome, which facilitated the presentation and binding of newly synthesized glycoproteins to the chaperone [25]. Dephosphorylation of the dog CLNX isoform on the homologous serine residue [25] had been shown to dissociate the protein from the ribosome uncoupling the protein synthesis machinery. Here, we demonstrate that CLNX is subject to dephosphorylation by CN under ER stress. This result is in agreement with Michalak's group [38], who recently found that CLNX deficient cells have constitutively active UPR. This has been suggested to represent an acute stress response [39]. We also show that another ER stressor, Tm, induced a small Ca^2+^ increase in the cytosol. These data are consistent with Tm-induced Ca^2+^ mobilization in fibroblast and CHO cells [40], [41] and suggest that both Tg and Tm are able to activate CN through a common and well characterized Ca^2+^/CaM dependent mechanism. We suggest that CN phosphatase activity provides the cell with additional time to restore ER homeostasis while the organelle is being refilled with Ca^2+^.

Surprisingly, we discovered that CN-A levels were significantly increased in the cytosol of *Xenopus* oocytes within 30–60 minutes of being stressed by Tg or DTT. We also found that CN-A interacted with PERK during stress, and that the kinetics of this association were correlated with the increase in CN-A levels. This suggested to us that the rise in CN levels could cause the subsequent interaction and activation of PERK. We confirmed this hypothesis in an independent model system, cultured mouse astrocytes. We found that ER-stress induced by OGD or thapsigargin treatment in cultured astrocytes rapidly increases CN-Aα levels. Because we were able to block this increase by siRNA treatments in astrocytes, it appears that stress-induced increases in CN-Aα are translationally dependent. Remarkably, the induction of the early UPR in astrocytes, as indicated by increased P-elF2α, was critically dependent on this rapid increase in CN-Aα. Specifically, when astrocytes were treated with siRNA specific for CN-Aα, we observed no significant increase in P-elF2α in response to Tg treatment. *In vitro* experiments with recombinant proteins also support this model. We demonstrated that the presence of CN-Aα/B significantly increased the autophosphorylation of GST-cPERK. A residual level of GST-cPERK phosphorylation in the absence of CN-Aα/B was likely due to dimerization of the GST-portion of GST-cPERK as reported by [42].

Another important observation from our pull-down experiments was that CN-A interacted with cPERK in a Ca^2+^ dependent manner. Association was significantly increased in conditions that mimic high cytosolic Ca^2+^. The significance of this finding is that this association should occur immediately after ER stress has been triggered when the cytosolic Ca^2+^ concentration initially increases. It is worth noting that in contrast to the CaM dependence of CN phosphatase activity, the association of CN with PERK appears to be inhibited by CaM. This interaction does not appear to be mediated by another protein, since no other protein was added to the *in vitro* assay. Moreover, we did not detect an interaction between CN-A and the catalytically inactive mutant GST-cPERK K/A. One explanation for this result is that lysine-618 is a critical residue in the CN binding site of PERK. Alternatively, CN may only be able to interact with PERK after a conformational change occurs in response to autophosphorylation. In this light, lysine 618 is critical to either PERK autophosphorylation and to the subsequent conformational change [8], [43]. In support of this, we and others [43] observe different mobilities for GST-cPERK and GST-cPERKK/A on SDS-PAGE (100 kD, and 85 kD, respectively), consistent with different protein conformations. We suggest that CN associates with PERK only after the kinase has been activated and once bound, stimulates further autophosphorylation of PERK.

Our model is consistent with the current of view of stress activated PERK. BiP is normally bound to the luminal domain of PERK and acts as negative regulator of activation [44]. In response to ER stress, BiP dissociates from its luminal domain of PERK to assist in luminal protein folding. This allows PERK oligomerization and its subsequent activation [9]. Our data take this stress activation sequence one step further by showing that CN-A binds to PERK and induces additional autophosphorylation at high cytosolic Ca^2+^. Interestingly, a ligand for PERK with the properties that we have described has been previously sought after [9]. We suggest that CN is a strong candidate for this ligand. Modulation of PERK activity by CN would represent a fine-tuning mechanism for optimal ER stress signaling. Moreover, CN/PERK interaction may constitute an example of at least partial dissociation from stress sensor activation, since ATF6, IRE1α and PERK would not all be activated by the same mechanism of titration from BiP. In this scenario, members of the proapoptotic Bcl-2 family, BAX and BAK [45], have been shown to interact with the cytosolic domain of IRE1α during ER stress [46]. It would appear that both IRE1α and PERK are actively regulated by cytoplasmic signals.

Another interesting finding was that PERK phosphorylated CN-A at resting concentrations of cytosolic Ca^2+^. Phosphorylation decreased the V_max_ of CN to 70%, without changing its affinity (K_m_) for substrate (Table S1). It is possible that phosphorylation of CN-A by PERK generates a more pronounced effect when CaM dissociates from CN upon Ca^2+^ decrease. This event could have more physiological relevance when ER Ca^2+^ homeostasis is being restored after stress by Ca^2+^ removal from the cytosol. Phosphorylation of CN has previously been observed *in vitro* by both CaM Kinase II and PKC [47], [48]. In all cases, phosphorylated CN exhibits less phosphatase activity. Interestingly, PERK phosphorylation was reduced at low Ca^2+^ concentration, when CN-A was phosphorylated. This appears unlikely to be the result of dephosphorylation by CN, since its phosphatase activity is significantly reduced in both low Ca^2+^ and when it is phosphorylated. The decrease of PERK phosphorylation is more likely a consequence of CN dissociating from PERK at low Ca^2+^ concentrations as suggested in Figure 3C.

Physiologically, we presented evidence suggesting that knock down of early CN levels with morpholinos increased the susceptibility of the *Xenopus* oocytes to undergo apoptosis. This suggested an important regulatory role of CN in preventing or delaying apoptosis during ER stress. This interpretation is in agreement with other reports suggesting that the susceptibility of cells to undergo apoptosis during stress depends on the amount of releasable Ca^2+^ from the ER [49], [50]. CN dependent dephosphorylation of CLNX, which increases SERCA 2b activity, is likely to minimize problems with protein folding during acute ER stress by rapidly restoring ER Ca^2+^ stores. At the same time, the interaction of CN with PERK would be expected to rescue cells from apoptosis by strongly attenuated new protein translation.

**Figure 8:**
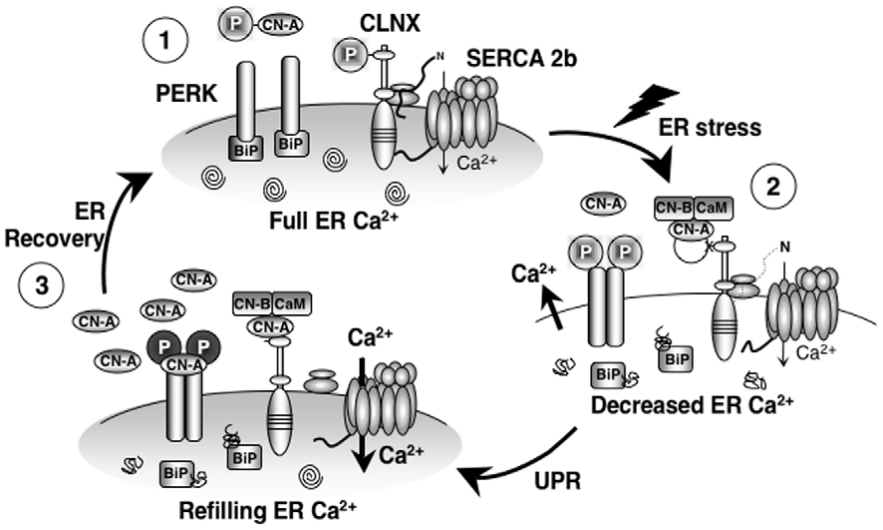
Role of CN in the early phases of ER stress. (**1**) Resting conditions of the ER: CLNX is phosphorylated, interacting with SERCA 2b and inhibiting its activity. CLNX is also interacting with the ribosome, increasing the capacity of protein folding. PERK is associated with BiP, which prevents its autophosphorylation. Protein processing and folding is optimal (depicted by spirals). (**2**) ER stress: unfolded proteins accumulate in the ER lumen, BiP dissociates from PERK, permitting its dimerization and autophosphorylation, which leads to attenuation of protein synthesis. At the same time, Ca^2+^ is released, activating CN, inducing dephosphorylation of CLNX, thereby removing pump inhibition. (**3**) CN levels are increased, leading to the association of CN with pre-activated PERK, which induces further PERK auto-phosphorylation, increasing the phosphorylation level of eIF2α. This emphasizes the protein translation inhibition. If cell Ca^2+^ levels are restored (**1**), CN becomes phosphorylated by PERK, decreasing its activity. CN expression also returns to resting levels further reducing its signaling. These steps, in combination with a full Ca^2+^ store and BiP re-association with PERK, restore normal protein translation and ER homeostasis.

In summary, this study reveals a novel role for CN at the initiation of the ER stress cascade. We have incorporated these mechanistic insights into a comprehensive model (Figure 8) that also accommodates findings related to PERK activation and CLNX-ribosome association as described by others [8], [9], [25]. Our discovery that CN activity plays an important role in the acute phase of ER Stress reveals an additional level of complexity to the UPR. It is important to distinguish this new role of CN during the early UPR from its distinct cell death function during later time points of the UPR. In particular, it has been reported that prolonged exposure of cells to CN inhibitors leads to upregulation of CHOP and subsequent apoptosis [51]. The new function of CN that we uncovered in this manuscript occurs at an earlier step in the UPR, prior to induction of CHOP. UPR has been implicated in a variety of cellular processes such as control of nutritional and differentiation programs [52]. It is also associated with numerous diseases like neurodegenerative disorders [53], cancer [54], viral infection [55] or ischemic injury [56]. Understanding the impact of CN activity in ER stress will yield new insights into the underlying causes of these physiological and pathological processes.

## Materials and Methods

### Vectors and Reagents

The *Xenopus* expression vectors for rat CLNX have previously been described [4]. The cDNAs encoding mouse PERK and the inactive kinase PERK K/A [32] were subcloned into pHN vector [57]. Fusion proteins corresponding to the cytosolic domain of PERK and PERK K/A in fusion with GST (GST-cPERK and GST-cPERK K/A) were generated by PCR and subcloned into pGEX-4T-2 (Amersham Biosciences, Piscataway, NJ). For GST-cPERK and its mutant, the forward primer was 5′-ACTGGAATTCCCATGCGCAGGCTTTTCCATCCTCAG and the reverse primer was 5′-ACTGCTCGAGCTAGTTGCCAGGCAGTGGGCTGTA using pHNb-PERK or pHNb-PERK K/A as templates. The PCR products were subcloned into EcoRI and XhoI sites. Automatic sequencing of all cDNA constructs was performed at the UTHSCSA core facility. All oligonucleotides and restriction enzymes were purchased from Invitrogen Life Technologies (Carlsbad, CA).

Unless otherwise specified, all chemicals were purchased from Sigma-Aldrich Corp. (St. Louis, MO). Stock solutions of Tg were resuspended in DMSO (2 mM) and stock solutions of Tm were resuspended in methanol at 45°C (5,000 µg/ml).

### *In Vitro* Transcription and Oocyte Protocols

CLNX mRNA was prepared as described previously [57].

### CN-Aα siRNA knockdown

C8D1A cells (ATCC; Manassas, VA; cat# CRL-2541) were plated at 1×10^5^ per well in a 6 well format prior to transfection. CN-Aα siRNA (PPP3CA) (Dharmacon; Lafayette, CO) was used at 100 nM and transfected with Dharmafect #4 (Dharmacon, Lafayette, CO; cat# T2004-02). Transfections were carried out following the manufactuers protocol. Astrocytes were transfected for 17 hours and observed 48 hours post transfection. At 48 hrs, astrocytes were subjected to 1 µM final DMSO vehicle or 1 µM thapsigargin treatment for 1 hour at 37°C. Astrocytes were then rinsed twice with PBS and scrapped into 100 µl of SDS sample buffer (62.5 mM Tris-HCl, pH 6.8, 2% w/v SDS, 10% glycerol, 50 mM dithiothreitol) and supplemented with 1 mM sodium orthovanadate (Sigma; St. Louis, MO), 10 units/ml Leupeptin (Sigma; St. Louis, MO), and 10 units/ml aprotinin (Sigma; St. Louis, MO) and used for Western blotting.

### Western blots, Immunoprecipitations and Co-immunoprecipitations

Oocytes extracts were prepared as described in [57] with the modification that the cytosolic fraction (supernatant) and/or the microsomal fraction (pellet) were separated for analysis. CLNX and CN-A were detected with antibodies from Assay Designs (Ann Arbor, MI, Cat #SPA-860 and Cat # C1956). PERK antibody was from ABGENT (San Diego, CA, Cat# AP8054b). P-eIF2α and BiP were detected with antibodies from Assay Designs (Ann Arbor, MI, Cat # KAP-CP131E and Cat# SPA-826). α-actin antibody was obtained from Santa Cruz Biotechnology, Inc. (Santa Cruz, CA). HRP-conjugated Donkey anti rabbit IgG and anti mouse IgG (Jackson ImmunoResearch Laboratories, Inc., West Grove, PA) were used as secondary antibodies. Oocyte immunoprecipitation (IP) of [γ-^32^]P-labeled CLNX w*as* performed as described previously [4]. For Co-immunoprecipitations, microsomal pellets were obtained and membrane proteins were extracted as described previously [4].

Cultured C8D1A astrocytes (ATCC; Manassas, VA; cat# CRL-2541) were rinsed twice with PBS and scraped down with a rubber policeman in the presence of 100 µl of lysis buffer per 35 mm dish used. Lysis Buffer was prepared as 15 mM Tris HCl, pH 7.6, 150 mM NaCl, 10% Glycerol, 1% TritonX 100, 1 mM EDTA supplemented with phosphatase inhibitors (5 mM NaF, 0.4 mM Na_3_VO_4_, 1 mMNaPPi, 0.1 mM ZnCl_2_, 1 mM NaMOb) and protease inhibitors (0.2 mM AEBSF, 10 µM Leupeptin, 1 uM Pepstatin A and 0.8 mM Benzmaidine. Cell lysis was achieved by passing the astrocyte suspension 10 times through a 1 ml syringe with a 25 G 5/8 needle and collected in an eppendorf tube. Nuclei and cell debri were removed by centrifugation at 1000 g for 10 minutes. Supernatant containing astrocyte cell extract was collected and retained for Western blots or Immunoprecipitations. Calcineurin A and P-eIF2α were detected with antibodies from Assay Designs (Ann Arbor, MI; cat# SPA-610 and cat# KAP-CP131E respectively). Actin was detected with a mouse monoclonal antibody (Millipore; Billerica, MA; cat# MAB 1501). PERK antibody was obtained from ABGENT (San Diego, CA, Catalog# AP8054b).

### Cytosolic Free Ca2+ Concentration Measurements

Ca^2+^ changes were measured in individual oocytes by fluorescence microscopy using the Ca^2+^ indicator Fura-2 (Invitrogen-Molecular Probes, Eugene, OR). Oocytes were injected with 50 nl Fura-2 salt (50 µM final concentration) for 20–30 min at 18°C until equilibration was reached. Measurements were performed in ND96 low Ca^2+^ (5 mM Hepes, pH 7.5, 96 mM NaCl, 2 mM KCl, 1 m MgCl_2_) at 18–20°C using a Nikon Eclipse TE 300 microscope with a 20×0.75 NA multi-immersion (water for our experiments) lens. Fura-2 was excited at a wavelength of 340 and 380 nm, and emitted fluorescence was collected via a 510 nm long pass filter, using a ORCA-ER charge-coupled video camera device (Hamamatsu Photonics, Hamamatsu, Japan). Frames were collected every 2 s and the 340/380 ratio was analyzed using Open Lab software (Improvision, Lexington, MA) following background subtraction. Ca^2+^ calibration of Fura-2 fluorescence was performed *in vitro* using the Ca^2+^ calibration Buffer Kit #2 (Invitrogen-Molecular Probes, Eugene, OR). An affinity constant of 200 nM was obtained and used to convert Fura-2 ratios into Ca^2+^ concentrations according to [58]. R_max_, R_min_ and S_b_/S_f_ were measured as 8.0785, 0.40001 and 8.7369, respectively.

### GST Fusion Protein Purification

The GST fusion protein purification was performed as described in [22]. To avoid the formation of inclusion bodies, bacteria expressing GST- cPERK K/A were incubated at 28°C and rotated at 300 rpm. Elution of bound protein and dialysis were performed according to [22].

### GST Pull-down Assays

Binding of human recombinant CN-Aα/B (2 pmoles) (EMD Bioscience, San Diego, CA) to GST-cPERK (0.40 nmoles) was performed at 4°C during 1 hour with over-end rotation. As controls, either GST alone or GST-cPERK K/A (0.40 nmoles), and when indicated CaM (2 pmoles), were used. Proteins were incubated and the washes were performed as previously described [22] with the exception that the buffers were supplemented with the corresponding free Ca^2+^ concentrations. The proteins were eluted by boiling in reducing Laemmli Buffer and were resolved through 12% SDS-PAGE using a mouse anti CN-A antibody and visualized by enhanced chemiluminescence. Ca^2+^ concentrations were calculated according to existing algorithms [59].

### In Vitro Kinase Assay

CN-Aα/B phosphorylation and autophosphorylation of cPERK were performed as follows. GST-cPERK, GST-cPERK K/A or GST alone (0.1 µM), were incubated for 3 min at 30°C in a 30 µl reaction mixture [20 mM Tris-Cl, pH 7.5, 100 mM NaCl, 10 mM MgCl_2_, 1.5 mM DTT, 0.5 mM EGTA, 1 mM NaOAc, 6% glycerol and 0.1 mM ATP, 50 µCi [γ^32^P]ATP (6,000 Ci/mmol, PerkinElmer Life Sciences, Inc., Boston, MA) in the presence of 2 free Ca^2+^ concentrations (30 nM or 1.4 µM). CaM at 1.6 µM was added as indicated. After 3 min, CN-Aα/B (1.7 µM) was added and incubated for 30 min. The reactions were stopped by boiling in reducing Laemmli Buffer and the proteins were resolved through 10% SDS-PAGE. The gel was fixed, dried, and proteins were visualized by autoradiography.

### Microinjection of Morpholino Antisense Oligos against CN-A

The morpholino antisense oligos: Morpho oligo1 (5′- TAGAGAAATCTGTGTGGGAAATGTC) and Morpho oligo 2 (5′-AGGCGATCAATTGACAGCTGCTTCT) against CN−A and the standard oligo morpholino were obtained from Gene Tools (Philomath, OR). Morpholinos to *Xenopus laevis* CN-A sequence were designed from accession #s BC049001, AB037146 and AF019569, dissolved in dH_2_O and injected into the oocytes at a final concentration of 5 µM.

### Pulse-labeling of Proteins

Groups of oocytes (10, each) previously injected or not with the corresponding morpholino, were starved for 30 min in a Methionine/Cysteine-free medium RPMI-1640 (diluted 33%, Sigma) and injected with a 50-nl bolus of [^35^S]Methionine-Cysteine (1175 Ci/mmol, 14 mCi, Perkin Elmer). After a 45 minutes period of incubation, oocytes were instantly frozen on dry ice. The total homogenate prepared as follows: oocytes were resuspended in 200 µl of Lysis Buffer (40 mM Tris-HCl, pH 7.5, 50 mM NaCl, 250 mM sucrose,10 mM MgCl_2_, 2 mM EDTA, 0.5 mM EGTA) supplemented protease inhibitors (800 µM benzamidine, 200 µM AEBSF, 20 µM Leupeptin, and 1 µM Pepstatin A), homogenized and centrifuged at 100 g. The corresponding supernatants were boiled in reducing Laemmli Buffer. The proteins (30 µl of each supernantant) were resolved through 10% SDS-PAGE. The gel was fixed, dried, and proteins were visualized by autoradiography.

### Cell-free Apoptosis in Xenopus laevis Oocytes Extracts

The cell-free apoptosis assay was carried out as originally described by [36] and as modified for *Xenopus* oocytes by Saelim et al. [37].

### Statistical Analysis

Statistical significance was determined by Student t-test, one-way ANOVA, or Tukey's Multiple Comparison Test as appropriate. One and two asterisks indicate statistical significance differences at p<0.05 or p<0.001, respectively. Error bars are expressed as SEM. The number (n) refers to the number of experimental, independent replicates.

## Supporting Information

Figure S1 Overexpressed (exogenous) CLNX exhibits higher levels of phosphorylation compared to endogenous CLNX. CLNX immunoprecipitations showing [γ-32P] ATP-labeled CLNX from Xenopus oocytes extracts (top). Immunoprecipitated proteins from control oocytes (injected with ddH20) (Cntrl) or from oocytes overexpressing CLNX (mRNA CLNX) were resolved through a 10% SDS-PAGE. Phosphorylated CLNX was visualized by autoradiography. Each lane corresponds to immunoprecipitated CLNX from 15 oocytes per group. (bottom) Western blots of CLNX were performed from the same oocyte extracts before IP. Note that endogenous levels of CLNX phosphorylation are relatively minor and obscured by exogenous phosphorylated CLNX.
(0.07 MB TIF)

Figure S2 Calcineurin inhibitors CsA and FK506 do not induce ER Stress. (A) Western blot showing phosphorylation levels of eIF2α and BiP from vehicle control oocytes (lane 1) or oocytes treated with CsA (200 nM) and FK506 (20 nM) for 16 hours (lane 2). Two oocyte equivalents were loaded per lane and proteins were resolved through 12% SDS-PAGE (P-elF2α) or 7% SDS-PAGE (BiP). P-eIF2α antibody used from Assay Designs (cat# KAP-CP131E). Actin Western blot is shown as loading control. BiP antibody used from Assay Designs (cat# SPA-826).(B) Histograms from 5 independent Western blots pooled from 3 different frogs with n = 15 oocytes per group. Intensity values were normalized with Actin and are represented as the mean ± SEM. ns indicates no statistical significance.
(0.08 MB TIF)

Figure S3 Levels of PERK and CN-A immunoprecipitated with anti-CN-A from oocyte cell extracts. (A) CN-A immunoprecipitation (IP) followed by PERK Western blot from control oocyte extracts (lane 1) or oocytes treated with 1 uM Tg for 15, 30 or 60 minutes (lanes 2, 3 and 4, respectively), or oocytes treated with DTT for 60 minutes and washed for 0, 20 or 60 minutes (lanes 5, 6 and 7, respectively). Immunoprecipitated proteins were resolved through 7% SDS-PAGE, transferred to nitrocellulose and probed for PERK with an antibody from ABGENT(cat# AP8054b). Note the presence of two dark bands around 150 kD (above and below 150 kD) corresponding possibly to P-PERK and PERK respectively in lanes from ER stress-induced treatment (lanes 2–6). Interestingly, the band corresponding to P-PERK is reduced in control oocyte extracts (lane 1) or in extracts from DTT treated/washed oocytes for 60 minutes (lane 7). (B) Nitrocellulose membrane shown in A was stripped and probed for CN-A with an antibody from Assay Designs (cat# SPA-610). Note the increase in CN immunoreactivity detected in extracts from ER-stress-induced oocytes (lanes 2–6), which is practically undetectable in control oocytes (lane 1) or DTT treated/washed oocytes for 60 minutes (lane 7). The darker band running at around 50 kD corresponds to detection of IgG from the immunoprecipitation.
(0.27 MB TIF)

Figure S4 Specificity of PERK binding to anti-PERK labeled beads and characterization of anti-PERK antibodies. (A) Commassie blue stained 7% SDS-PAGE gel loaded with Xenopus oocyte extracts that were obtained from a co-imunoprecipitation with an antibody against CN-A from Assay Designs (cat# SPA-610) (lanes 2 and 4) or incubated with protein A/G agarose beads alone (lanes 1 and 3). After Protein A/G agarose pellet was obtained, proteins were resolved through a 7% SDS-PAGE. (B) A fraction of the immunoprecipitated sample was loaded on the gel and transferred to nitrocellulose, probed with PERK antibody (ABGENT cat# AP8054b) and developed by autoradiography. Bands around the molecular weight of PERK (dashed area around 150 kD) were obtained from the immunoprecipitated samples (lanes 2 and 4). These bands are absent in the agarose beads controls (lanes 1 and 3) indicating no specific binding of PERK to the agarose beads. The bottom gel in B shows that protein was loaded in lanes 1 and 3, but did not contain an PERK immunoreactivity. The darker spots in lanes 2 and 4 most likely correspond to the immunoglobulin from the immunoprecipitates. Oo extr 1× and Ooc extr 2× corresponds to extracts from 15 and 30 oocytes, respectively. (C) Western Blot from mouse cultured astrocyte extracts (ATCC catalog # CRL-2541) at rest (Cntrl) or after 60 minutes of ER stress induction by oxygen glucose deprivation (OGD). Protein extracts were run on 7% SDS-PAGE and transferred to nitrocellulose membranes. Membrane on the left panel was probed with a PERK antibody generated in house (anti-PERKUT). Notice the presence of distinct bands around the molecular weight of PERK (dashed area around 150 kD). Right panel corresponds to a similar membrane probed with the anti-PERKUT antibody, in combination with the antigenic peptide used to generate the antibody. Notice the disappearance of bands at the molecular weight of PERK (dashed area around 150 kD) indicating competition of the antigenic peptide for PERK proteins present on the right membrane. The other bands remain unchanged in both membranes on top and bottom panels and are considered non-specific. (D) Western Blot probed with the Pre-immune serum and compared to the anti-PERKUT for primary mouse astrocyte extracts. Proteins were resolved through 7% SDS-PAGE and transferred to nitrocellulose membranes. Membrane probed with rabbit serum before immunization is non-reactive in comparison with middle panel membrane that was probed with anti-PERKUT antibody. Note again that the bands around 150 kD are competed off with the peptide used to immunize the rabbits. (E) Immunoprecipitation from primary mouse astrocytes using different concentrations of the generated anti-PERKUT antibody (lanes 2, 4 and 5) or pre-immune serum (lanes 1 and 3) followed by Western Blot with a commercial antibody from Abgent (cat# AP8054b), anti-PERKCM. Note the top band in the dashed rectangle is darker than the lower band at 150 kD and likely corresponds to P-PERK and PERK, respectively. P-PERK is known to migrate higher than PERK. (F) Western Blot from primary mouse astrocytes using pre immune serum (left panel) or anti-PERKUT antibody (middle panels) or commercial antibody anti-PERKCM (from Cell signaling cat#3179S). Extracts were obtained from cultured astrocytes at rest (lanes 1 and 3, Ctrl) and after 60 minutes OGD were used (lanes 2 and 4). Extracts from astrocytes were also treated with Vehicle (lane 5, Veh) and 300 nM thapsigargin (lane 6, Thap). Note the stress induced increase in PERK-P using the anti-PERKUT, but not the anti-PERKCM antibody.
(0.42 MB TIF)

Figure S5 Quantification of PERK loading for GST pull-down assay. Molar amounts of protein for GST-pull-down assays were calibrated by loading known volumes of proteins (GST, GST-cPERK and GST-cPERKK/A) side by side on the same gel with known volumes of Albumin (1 µg/µl) as a standard. Proteins were resolved through a 10% SDS-PAGE and stained with Coomassie blue. The concentration of GST, GST-cPERK and GST-cPERK K/A was converted to µmoles using the Albumin standard curve and apparent molecular weight of GST = 25 kD; GST-cPERK = 90 kD and GST-cPERKK/A = 75 kD.
(0.37 MB TIF)

Figure S6 Coomassie blue stained gel shown as loading control for the kinase assay on Figure 4A. Proteins were resolved through a 12% SDS-PAGE. Notice a distinct band at around 56 kD corresponding to CN-A only in lanes where CN-A/B has been added (lanes 1–6 and 8). Loading of cPERK is almost undetectable by Coomassie staining.
(0.12 MB TIF)

Figure S7 Phosphatase activity of CN-A. The specific activity of human recombinant CN-A was measured spectrophotometrically (O.D.410 nm) using p-NPP as substrate varying concentrations from 5 to 150 mM. The assay was initiated by addition of p-NPP, incubated at 30°C for 20 min and stopped by addition of 200 µl of 13% K2HPO4 and immediately chilled on ice. Specific activity was based on a pKa of 7.17 obtaining a measured molar extinction coefficient of 17,300 M-1 cm-1 at 410 nm at pH 8.58 for p-NPP. (A) The specific activity of CN-A without ATP (CN w/o ATP), without PERK (CN w/o PERK) compared to phosphorylated CN-A (P-CN) in high (3.2 µM) Ca2+. (B) Specific activity of CN-ATP, CN-PERK and P-CN in low (46 nM) Ca2+. Data are an average of 5 independent experiments. Error bars are within the size of the symbols (see Table S1).
(0.06 MB TIF)

Figure S8 [35S]-Methionine-Cysteine incorporation in oocytes injected with CN morpholinos and treated with Thapsigargin. (A) Autoradiography of total protein of total protein synthesized in control oocytes or injected with morpholinos as was described Figure 5, that has been untreated or exposed to Tg before a 45 minutes pulse label with [35S]-Methionine-Cysteine. (B) Histogram corresponding to densitometric analysis of total protein. Data are from three independent experiments (n = 3), * p<0.05.
(0.16 MB TIF)

Figure S9 Temporal progression of apoptotic-like morphology in isolated liver nuclei incubated with oocyte cytosolic extract. The percentage of apoptotic nuclei increases by 2 hours (first panels) and peaks by 4 hours (middle panels) for control extracts. In comparison, no change in the morphology of apoptotic nuclei is observed with buffer alone at 4 hours (right panels). Nuclei are stained with Hoechst dye (100 µg/µl) for visualization. Upper panels are high magnification of lower images for the white-framed regions.
(0.27 MB TIF)

Table S1 CN-A phosphatase activity.
(0.03 MB DOC)
