# Supplementary figures and images for: Calcineurin Interacts with PERK and Dephosphorylates Calnexin to Relieve ER Stress in Mammals and Frogs
Source: PLoS One. 2010 Aug 5;5(8):e11925. doi: 10.1371/journal.pone.0011925 (PMC2916823; doi:10.1371/journal.pone.0011925)

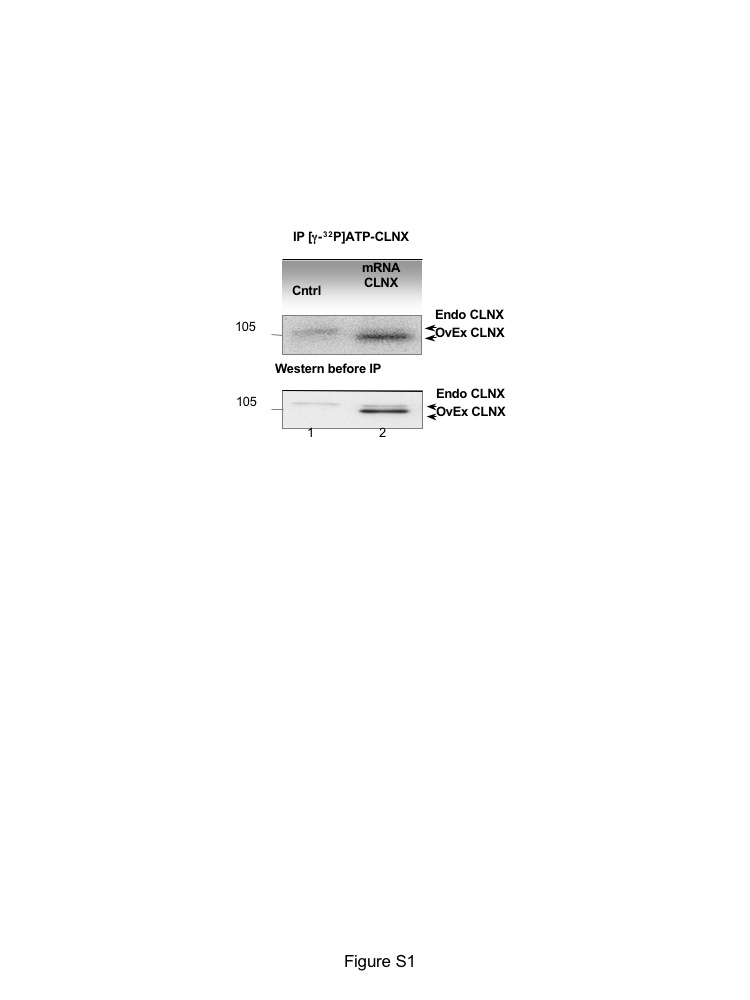

Supplement: Figure S1 — Overexpressed (exogenous) CLNX exhibits higher levels of phosphorylation compared to endogenous CLNX. CLNX immunoprecipitations showing [γ-32P] ATP-labeled CLNX from Xenopus oocytes extracts (top). Immunoprecipitated proteins from control oocytes (injected with ddH20) (Cntrl) or from oocytes overexpressing CLNX (mRNA CLNX) were resolved through a 10% SDS-PAGE. Phosphorylated CLNX was visualized by autoradiography. Each lane corresponds to immunoprecipitated CLNX from 15 oocytes per group. (bottom) Western blots of CLNX were performed from the same oocyte extracts before IP. Note that endogenous levels of CLNX phosphorylation are relatively minor and obscured by exogenous phosphorylated CLNX. (0.07 MB TIF) [file pone.0011925.s001.tif]

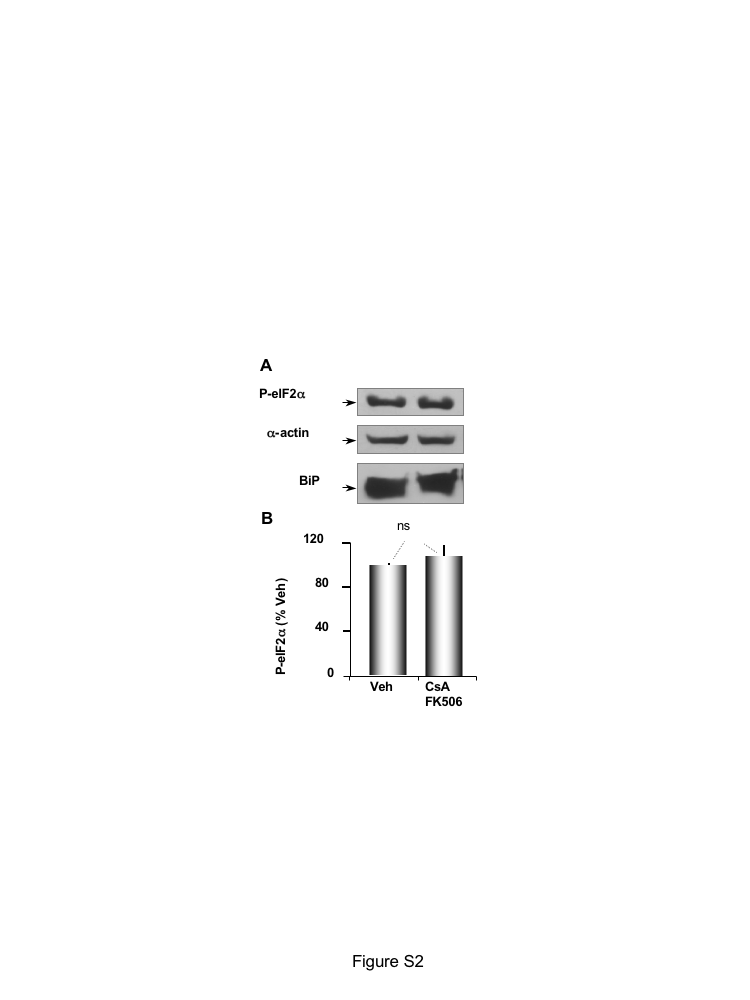

Supplement: Figure S2 — Calcineurin inhibitors CsA and FK506 do not induce ER Stress. (A) Western blot showing phosphorylation levels of eIF2α and BiP from vehicle control oocytes (lane 1) or oocytes treated with CsA (200 nM) and FK506 (20 nM) for 16 hours (lane 2). Two oocyte equivalents were loaded per lane and proteins were resolved through 12% SDS-PAGE (P-elF2α) or 7% SDS-PAGE (BiP). P-eIF2α antibody used from Assay Designs (cat# KAP-CP131E). Actin Western blot is shown as loading control. BiP antibody used from Assay Designs (cat# SPA-826).(B) Histograms from 5 independent Western blots pooled from 3 different frogs with n = 15 oocytes per group. Intensity values were normalized with Actin and are represented as the mean ± SEM. ns indicates no statistical significance. (0.08 MB TIF) [file pone.0011925.s002.tif]

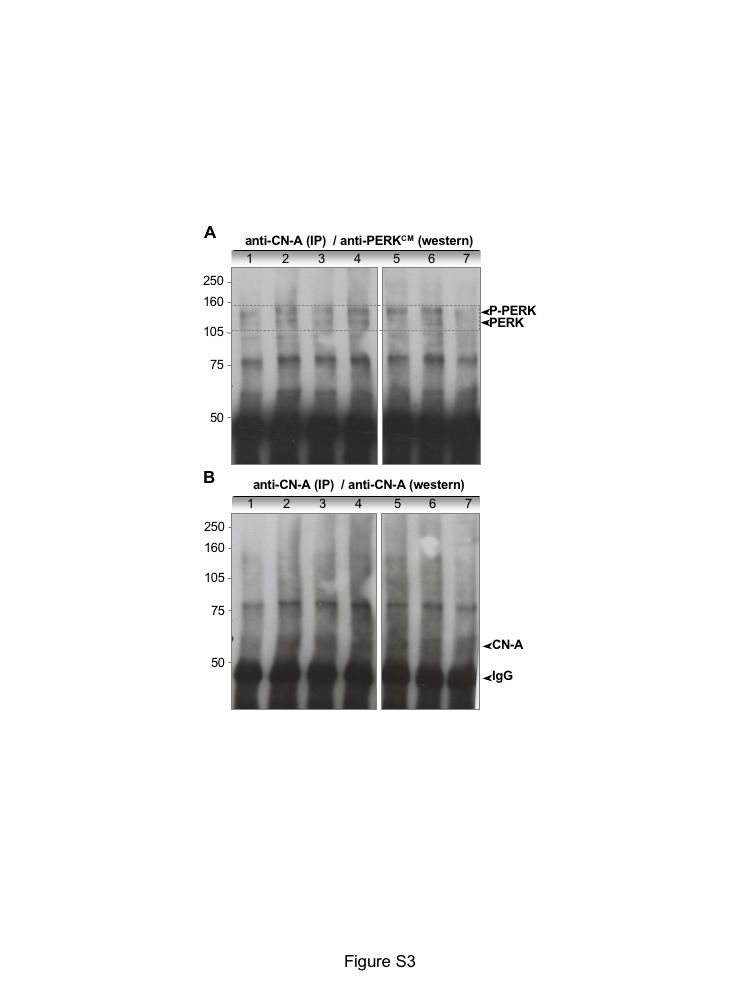

Supplement: Figure S3 — Levels of PERK and CN-A immunoprecipitated with anti-CN-A from oocyte cell extracts. (A) CN-A immunoprecipitation (IP) followed by PERK Western blot from control oocyte extracts (lane 1) or oocytes treated with 1 uM Tg for 15, 30 or 60 minutes (lanes 2, 3 and 4, respectively), or oocytes treated with DTT for 60 minutes and washed for 0, 20 or 60 minutes (lanes 5, 6 and 7, respectively). Immunoprecipitated proteins were resolved through 7% SDS-PAGE, transferred to nitrocellulose and probed for PERK with an antibody from ABGENT(cat# AP8054b). Note the presence of two dark bands around 150 kD (above and below 150 kD) corresponding possibly to P-PERK and PERK respectively in lanes from ER stress-induced treatment (lanes 2–6). Interestingly, the band corresponding to P-PERK is reduced in control oocyte extracts (lane 1) or in extracts from DTT treated/washed oocytes for 60 minutes (lane 7). (B) Nitrocellulose membrane shown in A was stripped and probed for CN-A with an antibody from Assay Designs (cat# SPA-610). Note the increase in CN immunoreactivity detected in extracts from ER-stress-induced oocytes (lanes 2–6), which is practically undetectable in control oocytes (lane 1) or DTT treated/washed oocytes for 60 minutes (lane 7). The darker band running at around 50 kD corresponds to detection of IgG from the immunoprecipitation. (0.27 MB TIF) [file pone.0011925.s003.tif]

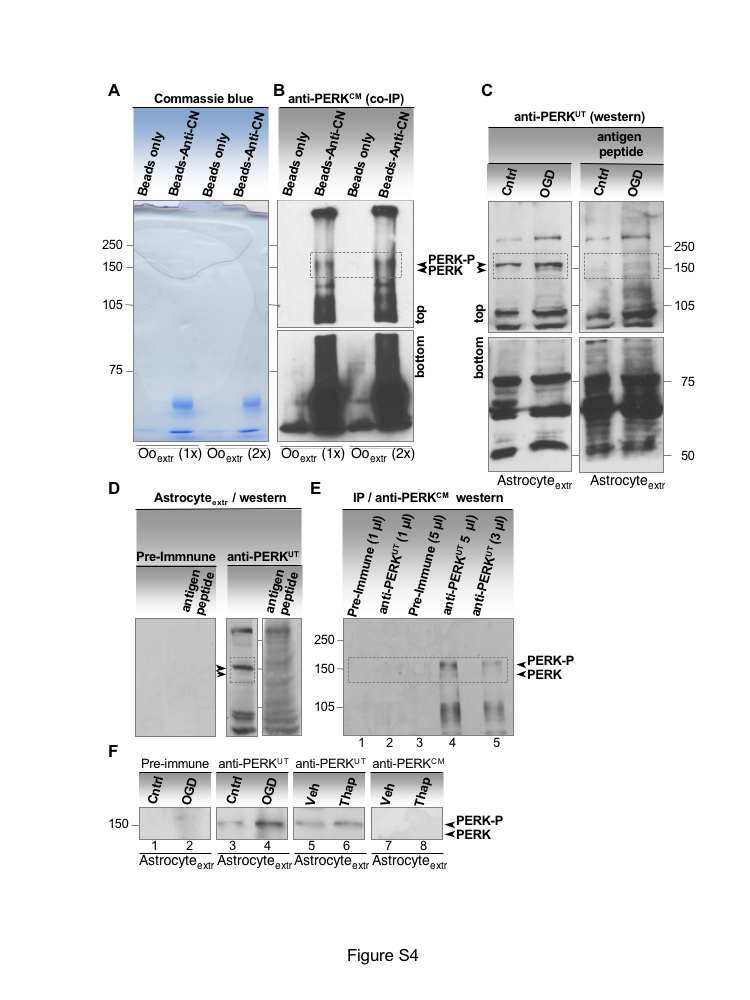

Supplement: Figure S4 — Specificity of PERK binding to anti-PERK labeled beads and characterization of anti-PERK antibodies. (A) Commassie blue stained 7% SDS-PAGE gel loaded with Xenopus oocyte extracts that were obtained from a co-imunoprecipitation with an antibody against CN-A from Assay Designs (cat# SPA-610) (lanes 2 and 4) or incubated with protein A/G agarose beads alone (lanes 1 and 3). After Protein A/G agarose pellet was obtained, proteins were resolved through a 7% SDS-PAGE. (B) A fraction of the immunoprecipitated sample was loaded on the gel and transferred to nitrocellulose, probed with PERK antibody (ABGENT cat# AP8054b) and developed by autoradiography. Bands around the molecular weight of PERK (dashed area around 150 kD) were obtained from the immunoprecipitated samples (lanes 2 and 4). These bands are absent in the agarose beads controls (lanes 1 and 3) indicating no specific binding of PERK to the agarose beads. The bottom gel in B shows that protein was loaded in lanes 1 and 3, but did not contain an PERK immunoreactivity. The darker spots in lanes 2 and 4 most likely correspond to the immunoglobulin from the immunoprecipitates. Oo extr 1× and Ooc extr 2× corresponds to extracts from 15 and 30 oocytes, respectively. (C) Western Blot from mouse cultured astrocyte extracts (ATCC catalog # CRL-2541) at rest (Cntrl) or after 60 minutes of ER stress induction by oxygen glucose deprivation (OGD). Protein extracts were run on 7% SDS-PAGE and transferred to nitrocellulose membranes. Membrane on the left panel was probed with a PERK antibody generated in house (anti-PERKUT). Notice the presence of distinct bands around the molecular weight of PERK (dashed area around 150 kD). Right panel corresponds to a similar membrane probed with the anti-PERKUT antibody, in combination with the antigenic peptide used to generate the antibody. Notice the disappearance of bands at the molecular weight of PERK (dashed area around 150 kD) indicating competition of the antigenic peptide for PERK [file pone.0011925.s004.tif]

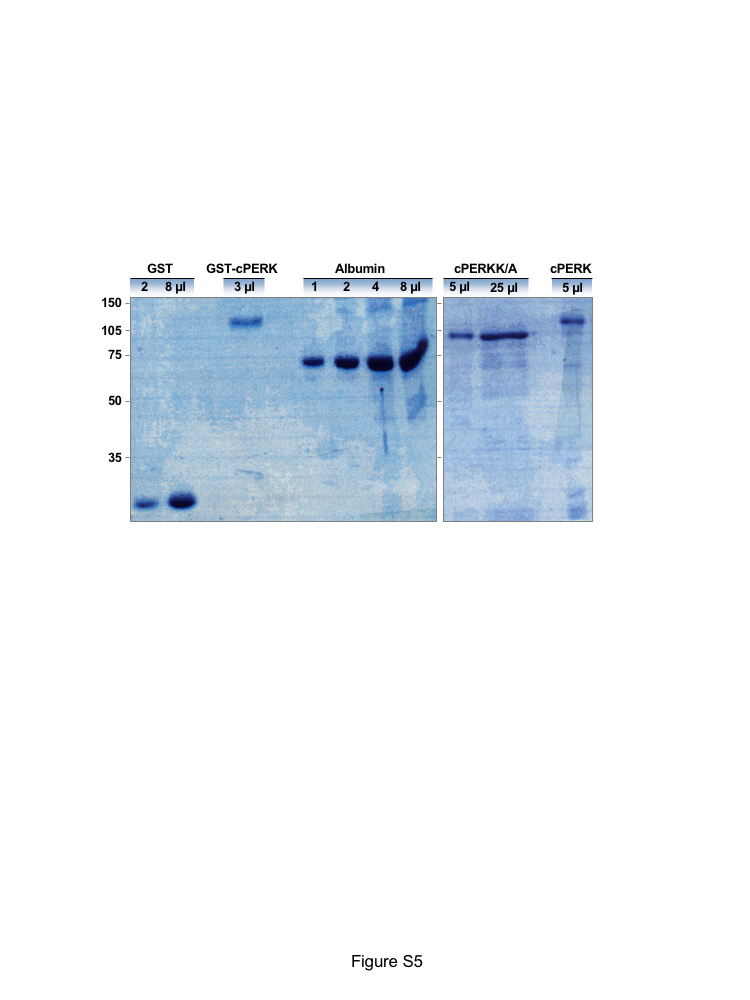

Supplement: Figure S5 — Quantification of PERK loading for GST pull-down assay. Molar amounts of protein for GST-pull-down assays were calibrated by loading known volumes of proteins (GST, GST-cPERK and GST-cPERKK/A) side by side on the same gel with known volumes of Albumin (1 µg/µl) as a standard. Proteins were resolved through a 10% SDS-PAGE and stained with Coomassie blue. The concentration of GST, GST-cPERK and GST-cPERK K/A was converted to µmoles using the Albumin standard curve and apparent molecular weight of GST = 25 kD; GST-cPERK = 90 kD and GST-cPERKK/A = 75 kD. (0.37 MB TIF) [file pone.0011925.s005.tif]

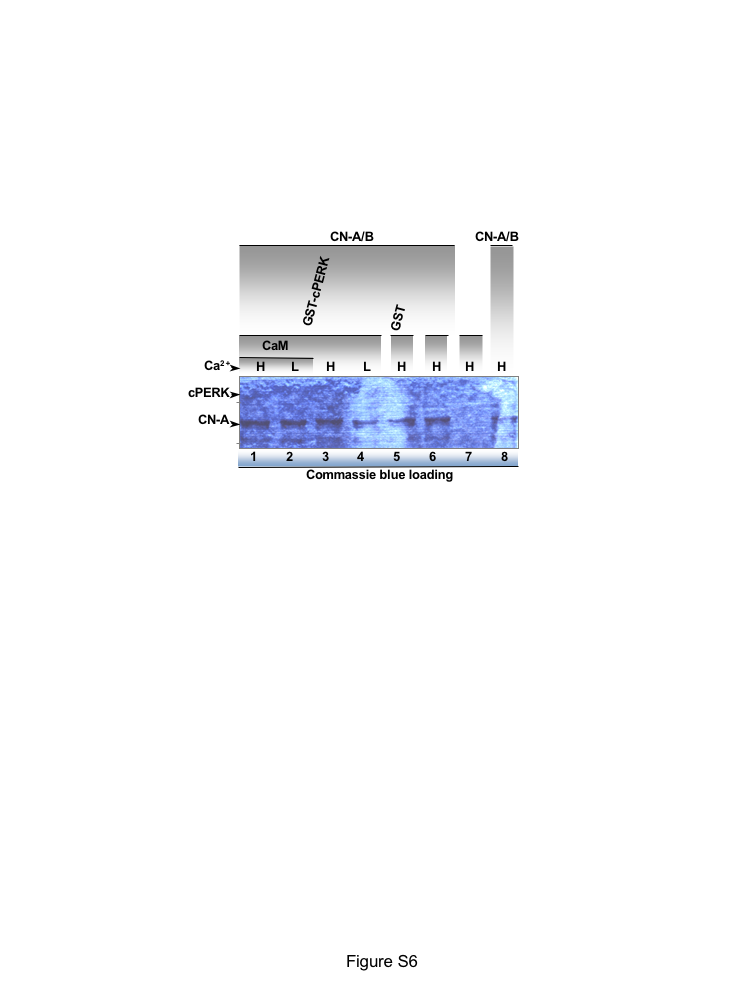

Supplement: Figure S6 — Coomassie blue stained gel shown as loading control for the kinase assay on Figure 4A. Proteins were resolved through a 12% SDS-PAGE. Notice a distinct band at around 56 kD corresponding to CN-A only in lanes where CN-A/B has been added (lanes 1–6 and 8). Loading of cPERK is almost undetectable by Coomassie staining. (0.12 MB TIF) [file pone.0011925.s006.tif]

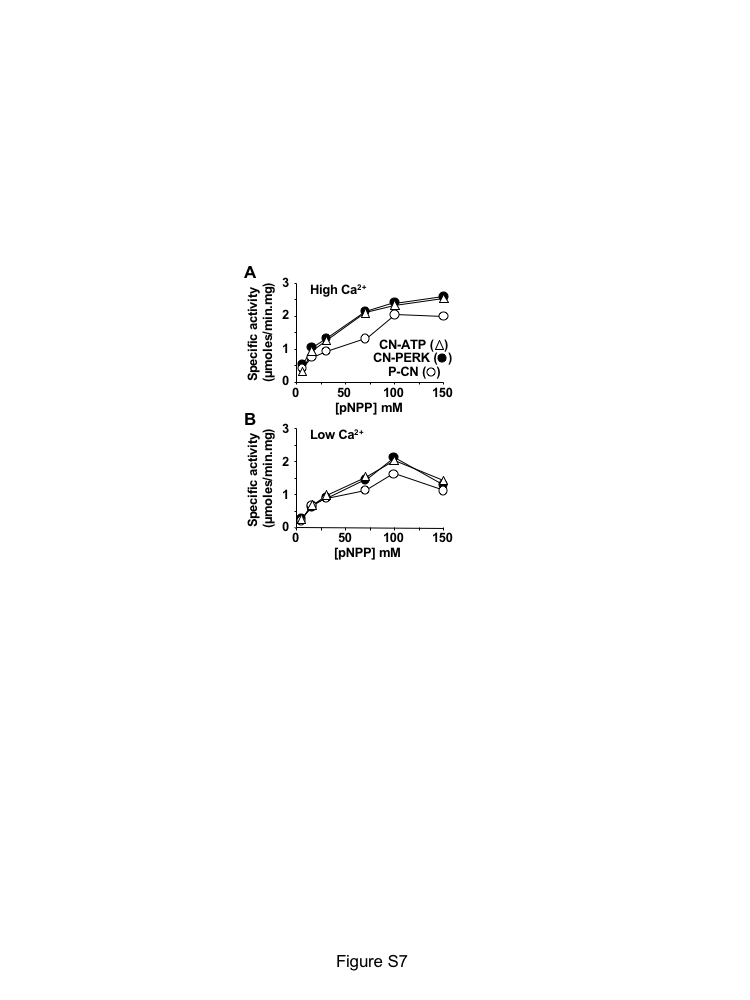

Supplement: Figure S7 — Phosphatase activity of CN-A. The specific activity of human recombinant CN-A was measured spectrophotometrically (O.D.410 nm) using p-NPP as substrate varying concentrations from 5 to 150 mM. The assay was initiated by addition of p-NPP, incubated at 30°C for 20 min and stopped by addition of 200 µl of 13% K2HPO4 and immediately chilled on ice. Specific activity was based on a pKa of 7.17 obtaining a measured molar extinction coefficient of 17,300 M-1 cm-1 at 410 nm at pH 8.58 for p-NPP. (A) The specific activity of CN-A without ATP (CN w/o ATP), without PERK (CN w/o PERK) compared to phosphorylated CN-A (P-CN) in high (3.2 µM) Ca2+. (B) Specific activity of CN-ATP, CN-PERK and P-CN in low (46 nM) Ca2+. Data are an average of 5 independent experiments. Error bars are within the size of the symbols (see Table S1). (0.06 MB TIF) [file pone.0011925.s007.tif]

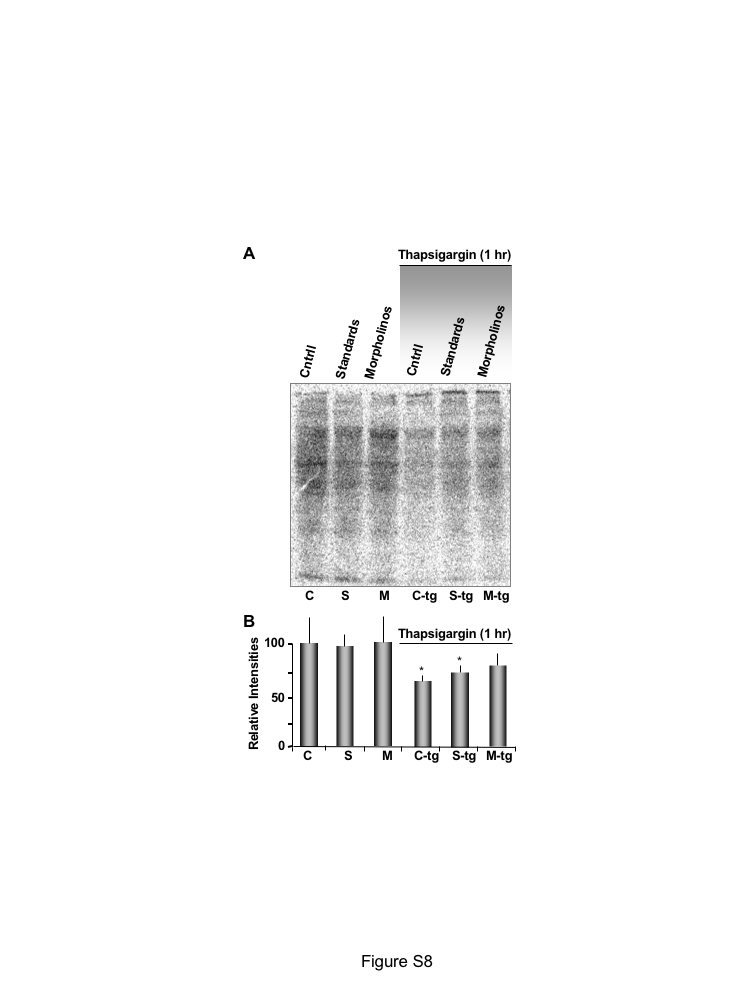

Supplement: Figure S8 — [35S]-Methionine-Cysteine incorporation in oocytes injected with CN morpholinos and treated with Thapsigargin. (A) Autoradiography of total protein of total protein synthesized in control oocytes or injected with morpholinos as was described Figure 5, that has been untreated or exposed to Tg before a 45 minutes pulse label with [35S]-Methionine-Cysteine. (B) Histogram corresponding to densitometric analysis of total protein. Data are from three independent experiments (n = 3), * p<0.05. (0.16 MB TIF) [file pone.0011925.s008.tif]

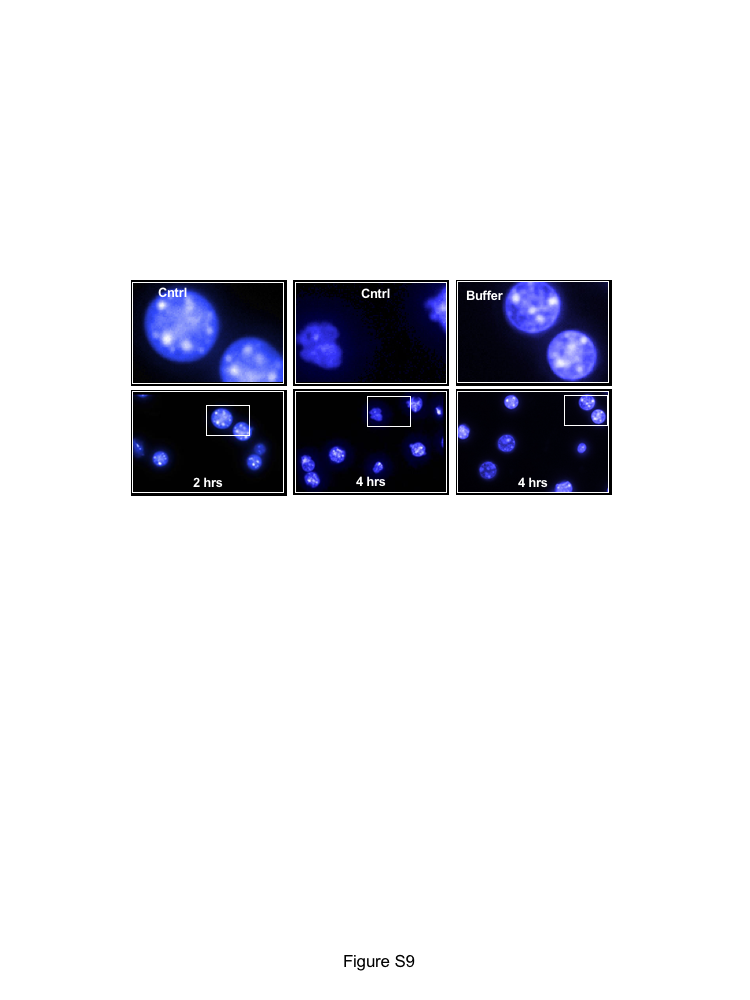

Supplement: Figure S9 — Temporal progression of apoptotic-like morphology in isolated liver nuclei incubated with oocyte cytosolic extract. The percentage of apoptotic nuclei increases by 2 hours (first panels) and peaks by 4 hours (middle panels) for control extracts. In comparison, no change in the morphology of apoptotic nuclei is observed with buffer alone at 4 hours (right panels). Nuclei are stained with Hoechst dye (100 µg/µl) for visualization. Upper panels are high magnification of lower images for the white-framed regions. (0.27 MB TIF) [file pone.0011925.s009.tif]
