# Supplementary material for: Calcineurin Interacts with PERK and Dephosphorylates Calnexin to Relieve ER Stress in Mammals and Frogs
Source: PLoS One. 2010 Aug 5;5(8):e11925. doi: 10.1371/journal.pone.0011925 (PMC2916823; doi:10.1371/journal.pone.0011925)
Supplement: Table S1 — CN-A phosphatase activity. (0.03 MB DOC) [file pone.0011925.s010.doc]

|  | **CN-A phosphatase activity** | | | | | | | | | | | |
| --- | --- | --- | --- | --- | --- | --- | --- | --- | --- | --- | --- | --- |
|  |  | Non-phosphorylated | | | | | |  |  | | Phosphorylated | |
|  | - PERK | | |  |  | - ATP | | | |  | | |
| Free [Ca^2+^] | Km (mM) | | Max. act. (mmol/min.mg) | Km (mM) | | | Max. act. (mmol/min.mg) | | Km (mM) | | | Max. act. (mmol/min.mg) |
| 1.2 μM | 24.0 (±6.0) | | 2.58 (±0.24) | 25.1 (±7.4) | | | 2.76 (±0.38) | | 26.0(±8.3) | | | 2.20 (±0.11) |
| 46 nM | 33.8 (±7.3) | | 2.22 (±0.15)* | 38.6 (±5.8) | | | 2.34 (±0.25) * | | 33.6 (±6.7) | | | 1.56 (±0.034) |

*p < 0.05 between phosphorylated and non-phosphorylated CN-A. Values are mean ± SEM (n = 5).

Table S1
